# Ubiquitination in lipid metabolism reprogramming: implications for pediatric solid tumors

**DOI:** 10.3389/fimmu.2025.1554311

**Published:** 2025-04-30

**Authors:** Weixin Zhang, Yile Xu, Yingjin Fang, Meng Li, Di Li, Huiqin Guo, Hang Li, Jing He, Lei Miao

**Affiliations:** ^1^ Department of Pediatric Surgery, Guangzhou Institute of Pediatrics, Guangdong Provincial Key Laboratory of Research in Structural Birth Defect Disease, Guangzhou Women and Children’s Medical Center, Guangzhou Medical University, Guangdong Provincial Clinical Research Center for Child Health, Guangzhou, Guangdong, China; ^2^ Department of Pancreatic Surgery, Fudan University Shanghai Cancer Center, Shanghai, China; ^3^ Department of Oncology, Shanghai Medical College, Fudan University, Shanghai, China; ^4^ Shanghai Key Laboratory of Precision Medicine for Pancreatic Cancer, Shanghai, China

**Keywords:** ubiquitin-proteasome system (UPS), lipid metabolism, pediatric solid tumor, cholesterol biosynthesis, fatty acid β-oxidation

## Abstract

Pediatric solid tumors represent a significant subset of childhood cancers, accounting for approximately 60% of new diagnoses. Despite advancements in therapeutic strategies, survival rates remain markedly disparate between high-income and resource-limited settings, underscoring the urgent need for novel and effective treatments. Lipid metabolic reprogramming is a fundamental hallmark of cancer, driving tumor progression, therapeutic resistance, and immune evasion through enhanced fatty acid uptake, increased *de novo* lipid synthesis, and activated fatty acid β-oxidation (FAO). Ubiquitination, a dynamic post-translational modification mediated by the ubiquitin-proteasome system (UPS), plays a crucial role in regulating lipid metabolism by modulating the stability and activity of key metabolic enzymes and transporters involved in cholesterol and fatty acid pathways. This review comprehensively examines the complex interplay between ubiquitination and lipid metabolic reprogramming in pediatric solid tumors. It delineates the mechanisms by which ubiquitination influences cholesterol biosynthesis, uptake, efflux, and fatty acid synthesis and oxidation, thereby facilitating tumor growth and survival. Furthermore, the review identifies potential UPS-mediated therapeutic targets and explores the feasibility of integrating ubiquitination-based strategies with existing treatments. By targeting the UPS to disrupt lipid metabolism pathways, novel therapeutic avenues may emerge to enhance treatment efficacy and overcome resistance in pediatric oncology. This synthesis of current knowledge aims to provide a foundation for the development of innovative, precision medicine approaches to improve clinical outcomes for children afflicted with solid tumors.

## 1 Introduction

Pediatric cancer, defined as malignancies in individuals under 19 years, poses a significant global health challenge due to its unique biological characteristics arising from developmental differences compared to adults (1–3). From 2018-2020, a research from China indicates that nearly 60% of newly diagnosed patients are solid tumor patients, followed by leukemia(30.56%) and lymphoma(10.06%) (4). Despite treatment advancements, five-year survival rates exceed 80% in high-income countries but drop below 30% in resource-limited settings, highlighting stark disparities in treatment access and the urgent need for novel therapies (5, 6).

Lipid metabolic reprogramming is a hallmark of cancer, driving tumor progression, therapeutic resistance, and immune evasion (7). Tumor cells exploit lipid metabolism by upregulating fatty acid uptake, enhancing *de novo* synthesis, and activating fatty acid β-oxidation (FAO) to meet their energy and biosynthetic demands (8–10). This dysregulated lipid metabolism also supports cancer cell survival in hypoxic and nutrient-deprived environments. Although FAO and the TCA cycle are typically oxygen-dependent, tumor cells adapt to hypoxia through metabolic reprogramming, including HIF-1α-mediated lipid uptake and storage, as well as alternative substrates such as short-chain fatty acids that may sustain FAO activity (10–16). Elevated *de novo* fatty acid synthesis and cholesterol levels further contribute to tumor progression, immune evasion, and drug resistance, making lipid metabolism a compelling therapeutic target (17–21). Moreover, aberrant lipid metabolism modulates immune cell functions within the tumor microenvironment; for example, a lipid-rich milieu can skew tumor-associated macrophages (TAMs) toward an immunosuppressive M2 phenotype (22–28) and impair T cell (29–31) antitumor activity via excessive fatty acid uptake that leads to ferroptosis and reduced expression of key cytotoxic molecules (32–37). In addition, enzymes involved in arachidonic acid metabolism—such as cytosolic phospholipase A2, cyclooxygenase, and lipoxygenase—have been linked to tumor resistance to radiotherapy (38). Targeting key lipid metabolic enzymes, including ACAT2 and SOAT, may help overcome these challenges and improve therapeutic outcomes (39).

The ubiquitin-proteasome system (UPS) is a key regulator of intracellular protein turnover and metabolic homeostasis. By governing the degradation of approximately 80% of intracellular proteins (40), ubiquitination orchestrates processes such as metabolic adaptation, tumor progression, and immune regulation (41–43). Recent studies reveal its crucial role in lipid metabolism, where ubiquitination of enzymes and transporters like CD36 modulates lipid uptake and utilization. Dysregulation of this system contributes to metabolic rewiring, fueling tumor growth (44–46).

This review explores the interplay between ubiquitination and lipid metabolic reprogramming in pediatric solid tumors. By examining how ubiquitination drives lipid metabolism and tumor progression, we identify potential therapeutic targets and discuss the integration of ubiquitination-based strategies with current treatments to disrupt tumor metabolism and improve outcomes in pediatric oncology.

## 2 Overview of ubiquitination

Ubiquitination is a fundamental post-translational modification that regulates protein stability, localization, and activity by attaching ubiquitin molecules to target proteins through a cascade of enzymatic reactions involving three core enzymes: E1 ubiquitin-activating enzymes, E2 ubiquitin-conjugating enzymes, and E3 ubiquitin ligases (47–49). Beyond its primary role in protein degradation, ubiquitination also governs various non-proteolytic processes, including the regulation of protein activity, subcellular localization, and protein complex formation (50). Notably, ubiquitin (Ub) chains linked through alternative residues (K6, K27, K33, K63, and M1) are predominantly involved in non-degradative functions, such as selective autophagy, DNA damage repair, and innate immunity (51, 52). Furthermore, ubiquitination plays a crucial role in maintaining DNA integrity and regulating gene expression, thereby facilitating the activation of diverse metabolic pathways in response to environmental changes (13). While comprehensive reviews, such as those by Liu (47) and Zhu (49), have extensively characterized the ubiquitination machinery and its roles in various cancers, their focus is primarily on adult malignancies, leaving the unique aspects of pediatric tumors largely unaddressed.”

In mammals, the ubiquitination system comprises two E1 enzymes, approximately 40 E2 enzymes, and over 600 E3 ligases, with E3 ligases conferring substrate specificity. E3 ligases are classified into three families based on their ubiquitin transfer mechanisms: RING (Really Interesting New Gene), HECT (Homologous to the E6AP Carboxyl Terminus), and RBR (RING-between-RING) ligases (53, 54).

Ubiquitination primarily determines protein fate through proteasomal or lysosomal degradation, with most ubiquitinated proteins being processed by the 26S proteasome. Deubiquitinating enzymes (DUBs) recycle ubiquitin molecules during this process. Beyond protein degradation, ubiquitination regulates diverse cellular processes, including protein trafficking, DNA repair, and signal transduction (55).

Dysregulated ubiquitination, driven by genetic or epigenetic aberrations such as mutations, amplifications, or deletions of ubiquitination-related genes, is strongly implicated in cancer (9). The functional outcomes of ubiquitination are influenced by the type of ubiquitin linkage and site of attachment, which affect processes like proteasomal degradation, protein activity, and subcellular localization. While lysine residues are the primary ubiquitination sites, other amino acids, such as N-terminal residues and cysteines, contribute to the complexity of ubiquitination’s regulatory roles (56).

The versatility of ubiquitination, driven by its diverse enzymes and linkages, is essential for maintaining cellular homeostasis and responding to stress. Its dysregulation in cancer underscores its potential as a promising therapeutic target.

## 3 Lipid metabolism reprogramming in tumors: distinct mechanisms in adult and pediatric cancers

Adult and pediatric tumors exhibit significant differences in biological characteristics, metabolic profiles, and nutritional intervention strategies. Adult tumors typically develop following long-term environmental exposures, lifestyle factors, chronic inflammation, and the gradual accumulation of genetic mutations, resulting in a high mutation burden and adaptive metabolic reprogramming. In contrast, pediatric tumors are more often driven by congenital genetic alterations and abnormal developmental signaling, leading to notable heterogeneity and distinct metabolic requirements—partly due to embryonic regulatory influences (57, 58).

### 3.1 Differences in lipid metabolism

#### 3.1.1 Differential dependency on fatty acid metabolism

Adult tumors generally rely on high levels of *de novo* fatty acid synthesis. For instance, cancers such as breast, prostate, and liver cancer commonly exhibit significant upregulation of fatty acid synthase (FASN) (59–61). In pediatric tumors, however, the reliance on fatty acid synthesis versus degradation varies by tumor type. For example, neuroblastoma(NB) primarily depends on FAO for energy metabolism (62), whereas medulloblastoma tends to favor lipid synthesis (63). Moreover, pediatric tumors may depend on alternative targets for exogenous lipid uptake. For instance, Fatty acid transport protein 2 (FATP2) plays a critical role in MYCN-amplified NB (64), whereas adult tumors more commonly depend on fatty acid transport proteins such as CD36 to enhance lipid absorption and metabolic reprogramming (65).

#### 3.1.2 Distinct regulation of cholesterol metabolism

Cholesterol metabolism plays a crucial role in both adult and pediatric tumors, though its regulation and clinical intervention strategies differ markedly. In adult tumors—such as prostate cancer and certain subtypes of breast cancer—upregulated cholesterol metabolism can promote cancer cell proliferation. Statins block HMG-CoA reductase and have been shown to slow disease progression in both precursor T-cell acute lymphoblastic leukemia and adult cancers like prostate and breast cancer (66, 67). In contrast, the application of statins in pediatric patients is generally limited due to safety concerns (68). In pediatric solid tumors, research on cholesterol metabolism remains sparse, and no specific clinical interventions targeting this pathway have been established, indicating a need for further exploration into its role in tumorigenesis and progression.

### 3.2 Impact of other regulatory factors and environmental influences

Recent studies suggest that the fatty acid-binding protein (FABP5) exerts age-dependent effects in tumor biology. In adult tumors, such as glioma, cervical cancer, and liver cancer, FABP5 promotes malignant transformation via activation of the NF-κB pathway (69–71). Conversely, in high-risk pediatric gliomas, elevated FABP5 levels have been associated with a more favorable prognosis, highlighting clear differences in regulatory mechanisms between age groups (72). Furthermore, dietary factors and obesity influence lipid metabolism differently across age groups. In adults, high-fat diets—particularly those rich in saturated and trans fats—are closely linked to colorectal, prostate, and liver cancers, underscoring the role of diet-induced metabolic dysregulation in tumor development (73–75). In the pediatric population, maternal high-fat diets during pregnancy may increase the risk of offspring developing leukemia and NB (76, 77), although some large-scale epidemiological studies have not confirmed a strong association (78). Additionally, despite children generally having lower fat reserves and muscle mass, their higher basal energy requirements render their lipid metabolic balance more vulnerable (79). Certain cancer treatments may also elevate triglyceride and free cholesterol levels, potentially increasing long-term health risks and recurrence probabilities (79–82).

### 3.3 The role of MYCN in lipid metabolic reprogramming

In NB, the proto-oncogene MYCN serves as a key driver of tumorigenesis by regulating the expression of multiple proteins involved in lipid metabolism, including FASN, stearoyl-CoA desaturase 1 (SCD1), and FATP2. These targets are particularly critical in MYCN- or MYC-driven tumors, while they may not be the primary oncogenic regulators in other cancer types (64).

In summary, the regulation of lipid metabolism varies markedly between tumors arising in different age groups. This heterogeneity reflects distinct underlying molecular biology and metabolic needs, emphasizing the importance of developing age-specific metabolic intervention strategies in future precision oncology.

## 4 Cholesterol metabolism in pediatric tumors

Cholesterol metabolism plays a critical role in pediatric tumor progression by supporting rapid cell proliferation (83), immune evasion (84, 85), and therapy resistance (86). Beyond serving as a structural component of cellular membranes, cholesterol mediates key signaling pathways that protect tumor cells from lipid peroxidation and enhance their metabolic adaptability within the tumor microenvironment (87). Additionally, cholesterol accumulation promotes immune suppression by altering dendritic cell maturation and dampening anti-tumor immune responses (88).

Cholesterol biosynthesis is essential for tumor growth and survival in pediatric cancers. Elevated cholesterol levels are linked to poor prognosis in NB (89). Moreover, combining caffeine treatment or FOXM1 inhibition with statins enhances the anti-tumor efficacy of statins against NB (90). Similarly, enhanced cholesterol biosynthesis drives tumor progression and oncogenic signaling, such as Hedgehog pathway activation, in Wilms’ tumor and medulloblastoma (91, 92).

**Figure 1:**
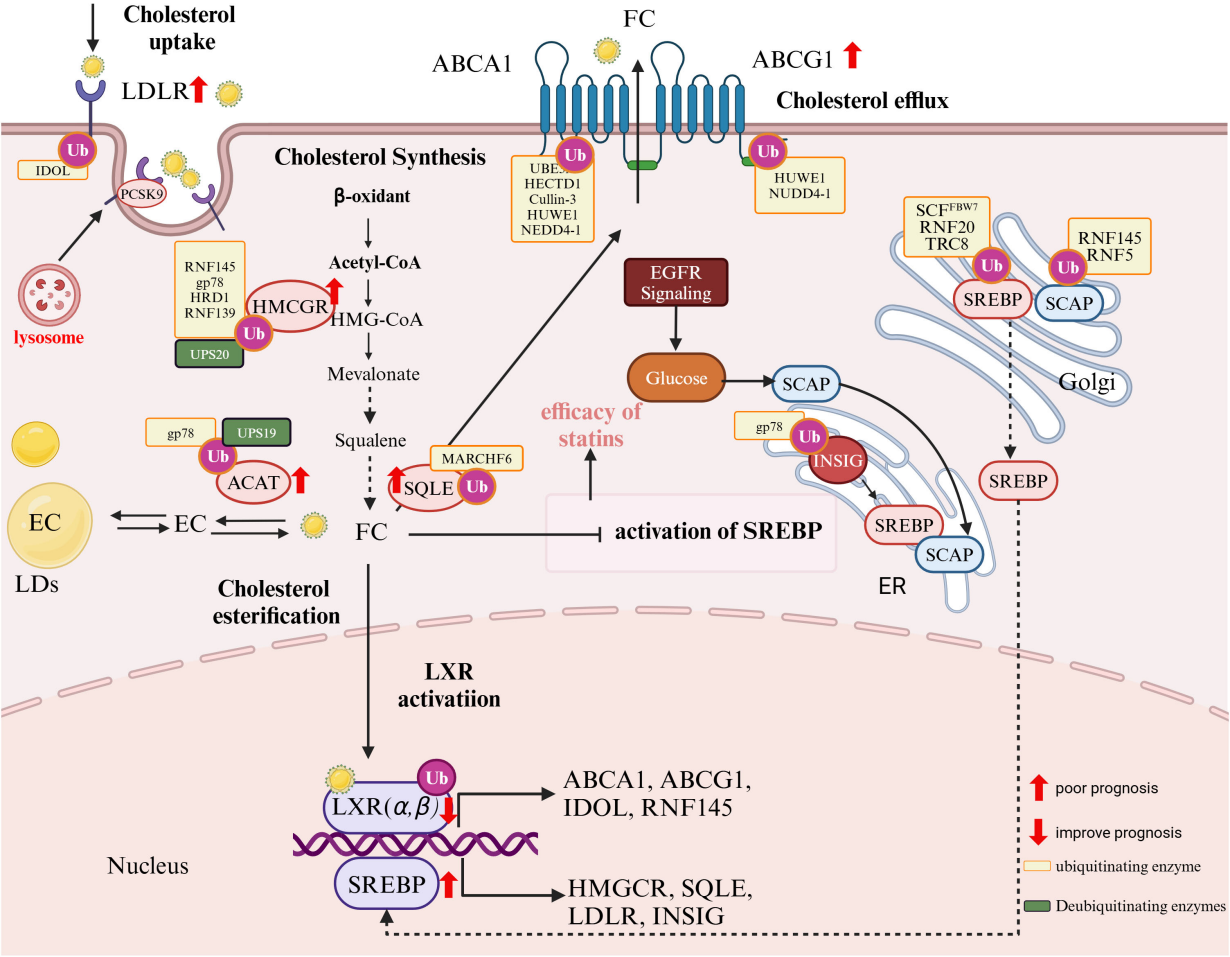
Ubiquitination and fatty acid (FA) metabolism in pediatric cancers. (Created in https://BioRender.com.) FA metabolism is driven by key enzymes and regulators, many of which are subject to ubiquitination and deubiquitination, marked in orange and green boxes, respectively. Sterol regulatory element-binding protein 1 (SREBP1) plays a central role in regulating FA synthesis. It is ubiquitinated by the Skp1-Cul1-FBW7 (SCF^FBW7) E3 ligase complex and Ring Finger Protein 20 (RNF20). Stabilized expression of SREBP1 promotes tumor progression. FA also activates peroxisome proliferator-activated receptors (PPARs), which are fine-tuned by UPS. PPAR activation induces the expression of cluster of differentiation 36 (CD36), a receptor involved in FA uptake. CD36, regulated by PARKIN-dependent ubiquitination, is associated with tumor progression, immune evasion, and metastasis in pediatric cancers, correlating with poor prognosis. However, the TSP-1/CD36 axis can inhibit angiogenesis under certain conditions. Internalized free fatty acids (FFAs) are converted into monoglycerides (MGs), diglycerides (DGs), and triglycerides (TGs), which are stored in lipid droplets (LDs). Upon energy demand, TGs are hydrolyzed by adipose triglyceride lipase (ATGL), which is ubiquitinated by COP1 and RNF213. FFAs can also be used to generate unsaturated fatty acids (e.g., MUFAs and PUFAs), a process requiring carnitine palmitoyltransferase 2 (CPT2), ATP-citrate lyase (ACLY), acetyl-CoA carboxylase (ACC), fatty acid synthase (FASN), stearoyl-CoA desaturase 1 (SCD1), and fatty acid-binding proteins FABP4 and FABP5. CPT2 is essential for FA transport into mitochondria. These enzymes (CPT2, ACLY, ACC, FASN, SCD1, FABP4, and FABP5) are frequently overexpressed in pediatric cancers and are drivers of tumor progression and poor prognosis.

The UPS plays a key role in this regulatory network by modulating the stability of proteins involved in cholesterol metabolism. When intracellular cholesterol levels are high, the UPS promotes the ubiquitination and subsequent degradation of proteins responsible for cholesterol biosynthesis and uptake, while the ubiquitination of proteins involved in cholesterol efflux is reduced to maintain adequate export function. Conversely, when cholesterol levels decrease, the UPS reduces the ubiquitination of biosynthetic and uptake proteins—thereby prolonging their half-life—while enhancing the ubiquitination of efflux transporters to limit cholesterol loss (93). In this way, the UPS tightly regulates cholesterol metabolism, and dysregulation of this system contributes to the metabolic reprogramming observed in pediatric tumors, involving specific E3 ligases and deubiquitinases (**Figure 1**) (94).

### 4.1 Transcriptional regulation of cholesterol metabolism in pediatric solid tumors

In pediatric solid tumors, cholesterol metabolism is tightly regulated by two key transcription factors: sterol regulatory element-binding protein 2 (SREBP2) and liver X receptors (LXRs). SREBP2 primarily drives cholesterol biosynthesis and uptake, while LXRs promote cholesterol efflux by inducing genes such as ABCA1, ABCG1, and APOE. Their activities are interlinked in a feedback loop that maintains cholesterol homeostasis; under high intracellular cholesterol conditions, LXRs can antagonize SREBP2 activity to prevent excessive cholesterol accumulation. Disruption of this balance—either through hyperactivation of SREBP2 or insufficient LXR-mediated clearance—leads to cholesterol accumulation that supports tumor growth, membrane synthesis, and oncogenic signaling.

### 4.2 The SREBP axis: sculpting tumor cholesterol homeostasis through UPS fine-tuning

SREBP2 plays a critical role in tumor cholesterol metabolism, driving proliferation and survival by inducing the expression of mevalonate pathway genes to enhance cholesterol and isoprenoid synthesis (95). Normally, the SCAP-SREBP2 complex is retained in the ER by insulin-inducible gene protein (Insig), preventing activation. Tumor cells bypass this regulation through mechanisms such as EGFR-induced the SREBP cleavage-activating protein (SCAP) glycosylation, enabling SREBP2 activation and increased lipogenesis (96). This dysregulation supports rapid proliferation and metabolic adaptability in glioblastoma, hepatocellular carcinoma, and NB. Inhibiting SREBP2 enhances statin efficacy and overcomes drug resistance, highlighting its therapeutic potential in pediatric tumors (90, 97, 98).

The UPS imposes an additional layer of control on SREBP2. The E3 ligase translocation in renal cancer from chromosome 8 (TRC8), initially identified as a tumor suppressor in renal cell carcinoma (99), inhibits tumor growth by regulating SREBP activation, likely through blocking ER-to-Golgi transport or promoting precursor degradation (100, 101). Similarly, the ring finger protein 145 (RNF145) targets SCAP for ubiquitination, limiting SREBP2 activation and cholesterol biosynthesis (102). gp78, another key E3 ligase, targets HMG-CoA reductase, a rate-limiting enzyme in the mevalonate pathway, for degradation to maintain cholesterol homeostasis (103). This differential regulation highlights the nuanced interplay between UPS components in balancing cholesterol synthesis and efflux.

Insig proteins further modulate this process. Insig-1, which is ubiquitinated and rapidly degraded, facilitates SREBP2 activation, while Insig-2 resists degradation under oxidative stress, protecting against lipid overload (104, 105). This interplay between SCAP, Insig proteins, and UPS ensures a balance between cholesterol synthesis and efflux.

Deubiquitination fine-tunes SREBP2 activity. Ubiquitin-specific protease 28 (USP28) stabilizes the active nuclear form of SREBP2 by removing ubiquitin chains, enhancing the expression of mevalonate pathway enzymes and supporting tumor metabolic demands. This dynamic regulation allows tumor cells to adapt to the metabolic requirements of growth and survival (106).

### 4.3 LXRs: cholesterol-sensing nuclear receptors in tumor metabolism

LXRs function as cholesterol sensors and are central to maintaining cholesterol homeostasis. Upon activation, LXRs induce the transcription of cholesterol efflux genes, including ATP-binding cassette transporter A1(ABCA1), ATP binding cassette transporter G1 (ABCG1), and apolipoprotein E (APOE), which reduce intracellular cholesterol levels. LXRs also regulate cholesterol uptake by inducing E3 ligases such as low-density lipoprotein (LDL) receptor (IDOL) (107) and RNF145, which ubiquitinate and degrade the low-density lipoprotein receptor (LDLR) to prevent cholesterol overaccumulation (108, 109).

In pediatric tumors, LXRs exhibit context-specific roles that influence tumor progression and prognosis. In NB, LXR activation upregulates ABCG1, promoting cholesterol efflux, protecting cells from oxidative stress, and enhancing survival (110). Conversely, in osteosarcoma(OS), LXRα functions as a tumor suppressor, with its activation reducing proliferation and sensitizing tumors to chemotherapy through mTOR signaling modulation. Combining LXR agonists with chemotherapeutics such as doxorubicin has shown to enhance efficacy and overcome chemoresistance (111).

The UPS adds another layer of regulation to LXR activity. SIRT1-mediated deacetylation increases LXR ubiquitination and turnover, altering its transcriptional activity in a context-dependent manner (112). However, the roles of specific E3 ligases or DUBs in regulating LXRs remain unclear.

### 4.4 Cholesterol uptake: LDL receptor pathway and its dysregulation

Cholesterol uptake is essential for tumor cells to meet their increased energy and biosynthetic demands. LDLR family mediates cholesterol endocytosis, playing a central role in lipid homeostasis. In pediatric tumors like osteosarcoma, LR3, a member of the LDLR family, promotes tumor proliferation and invasion, underscoring the role of LDLR signaling in metabolic reprogramming (113). Targeting the mTOR-LDLR axis with agents such as rapamycin has been shown to restore LDLR regulation and suppress tumor growth by limiting cholesterol uptake (114).

LDLR expression is tightly controlled by intracellular cholesterol levels. SREBP2 upregulates LDLR during cholesterol scarcity, enhancing cholesterol uptake, while LXRs induce the E3 ligase IDOL (Inducible Degrader of LDLR) under high cholesterol conditions, targeting LDLR for ubiquitination and lysosomal degradation (115–117).In pediatric tumors, this balance is often disrupted. Pathways such as mTOR activation bypass the LXR-IDOL axis, leading to persistent LDLR expression and excessive cholesterol uptake. In osteosarcoma, sustained LDLR activity supports tumor growth and metastasis (114).

Beyond transcriptional control, LDLR levels are modulated post-translationally. Proprotein convertase subtilisin/kexin type 9 (PCSK9) binds to LDLR and directs it to lysosomal degradation, exacerbating LDLR dysregulation in tumors (107, 118, 119). In pediatric cancers such as medulloblastoma, PCSK9 acts as a tumor-specific neoantigen, triggering T-cell responses and presenting potential as an immunotherapy target (120). Elevated PCSK9 expression has been associated with poor prognosis, drug resistance, and reduced progression-free survival (PFS) in certain cancers, highlighting its dual role in cancer progression and immune modulation (84, 121).

### 4.5 Cholesterol biosynthesis: from acetyl-CoA to cholesterol

Cholesterol biosynthesis is a tightly regulated pathway essential for tumor growth and metabolic reprogramming. Key enzymes, including HMGCR (3-hydroxy-3-methylglutaryl-CoA reductase) and squalene monooxygenase (SQLE), play central roles in maintaining cholesterol homeostasis and supporting cancer cell proliferation.

#### 4.5.1 HMGCR: a key regulator in cholesterol biosynthesis

HMGCR, the rate-limiting enzyme of cholesterol biosynthesis, is extensively regulated by UPS. E3 ligases such as RNF145, gp78, and HRD1 promote HMGCR degradation, while ubiquitin-specific protease 20 (USP20) stabilizes HMGCR, enhancing cholesterol synthesis and driving tumor growth (122–126). Dysregulation of this balance, such as reduced RNF139 activity, results in HMGCR overexpression, contributing to drug resistance and tumor progression.

Clinically, HMGCR overexpression is frequently observed in pediatric cancers such as NB, rhabdomyosarcoma, and osteosarcoma, where it promotes proliferation, invasion, and survival while reducing therapy efficacy (127–130). Statins, including lovastatin, inhibit HMGCR, blocking cholesterol biosynthesis, inducing apoptosis, and sensitizing tumor cells to chemotherapy and radiotherapy. Statins also target the mevalonate pathway, disrupting Ras/Rho protein prenylation and enhancing antitumor immunity (131, 132).

#### 4.5.2 Squalene monooxygenase

SQLE catalyzes a critical step in cholesterol biosynthesis and is strongly associated with chemoresistance and poor prognosis in cancers, including osteosarcoma. Inhibitors like FR194738 deplete intracellular cholesterol, suppress FAK/PI3K/Akt/mTOR signaling, reduce malignancy, and restore chemosensitivity (133–135).

Beyond its role in cholesterol biosynthesis, SQLE plays a pivotal role in regulating ferroptosis and cuproptosis, modulating the tumor microenvironment and potentially enhancing immunotherapy efficacy (134). Ferroptosis, characterized by iron-dependent lipid peroxidation and reduced glutathione peroxidase 4(GPX4) activity, leads to oxidative damage and cell death (136). QLE promotes ferroptosis by enhancing reactive oxygen species (ROS) production and lipid peroxidation. Cuproptosis, a form of cell death triggered by copper toxicity, disrupts the TCA cycle via protein–lipid interactions in mitochondria, leading to protein aggregation and cell death (134, 137–140). SQLE may regulate cuproptosis by modulating intracellular copper metabolism and mitochondrial function.

Like HMGCR, SQLE is regulated by the UPS via the E3 ligase Membrane-associated ring-CH-type finger 6 (MARCHF6), which promotes its degradation under conditions of elevated cholesterol. MARCHF6 also regulates other enzymes in the cholesterol biosynthetic pathway, including DHCR24 and LDM, highlighting the UPS’s central role in maintaining cholesterol homeostasis. Cholesterol enhances MARCHF6 activity to degrade SQLE, whereas unsaturated fatty acids inhibit this process by disrupting their interaction (141–143).

### 4.6 Cholesterol efflux: transport and removal mechanisms

Cholesterol efflux is critical for maintaining cellular cholesterol balance, preventing cytotoxic accumulation, and supporting cellular integrity. ATP-binding cassette (ABC) transporters, particularly ABCA1 and ABCG1, mediate cholesterol efflux to apolipoproteins and HDL, enabling reverse cholesterol transport (144). Dysregulated cholesterol efflux in pediatric tumors drives metabolic reprogramming, therapy resistance, and immune evasion.

#### 4.6.1 ABCA1 and ABCG1: key transporters in cholesterol homeostasis

ABCA1 and ABCG1 exhibit context-specific roles in pediatric cancers. ATP-binding cassette subfamily B member 1 (ABCB1) effluxes doxorubicin, reducing its intracellular accumulation and diminishing both its toxicity and the immunogenic cell death it induces, thereby contributing to chemoresistance in osteosarcoma (145). In contrast, ABCA1 expels isopentenyl pyrophosphate (IPP), a molecule that activates anti-tumor Vγ9Vδ2 T cells. Enhancing ABCA1-dependent IPP efflux may help overcome ABCB1-mediated resistance (145). Conversely, in medulloblastoma, ABCA1 overexpression confers radiation resistance, likely by altering cholesterol efflux and membrane lipid composition, which could affect DNA repair and stress response pathways (146). Although the molecular basis underlying this discrepancy remains to be fully elucidated, these differences may reflect tumor-specific downstream signaling or microenvironmental factors that modulate ABCA1 function. In NB, ABCA1-mediated cholesterol efflux protects tumor cells from oxidative stress, facilitating survival under metabolic stress (110).

ABCG1 supports cancer stem-like cells (CSCs) in osteosarcoma and Ewing sarcoma, enhancing cholesterol efflux to sustain CSC viability and multidrug resistance. In NB, the LXR-ABCG1 axis protects tumor cells from oxidative damage caused by oxidized cholesterol. Targeting ABCG1 in CSCs could reduce tumor recurrence and improve therapeutic outcomes (110, 147).

UPS tightly regulates ABCA1 and ABCG1 by modulating their degradation and stability. E3 ligases such as HUWE1, NEDD4-1, and HECTD1 ubiquitinate these transporters, reducing their cholesterol efflux capacity and contributing to metabolic dysregulation (148, 149). Additionally, ubiquitin-protein E3 ligase A (UBE3A)-mediated ubiquitination of ABCA1 has been implicated in myelin-induced foam cell formation in models of CNS repair (M. Loix et al., unpublished). Furthermore, Cullin-3, a cullin-RING ubiquitin E3 ligase, is activated through thrombin–protease-activated receptor (PAR1) signaling, leading to the degradation of ABCA1 and further disrupting cholesterol homeostasis (150, 151). Activation of Cullin-3 requires neddylation, a post-translational modification in which the ubiquitin-like protein NEDD8 conjugates to cullins, enhancing their ubiquitin ligase activity (152). The COP9 signalosome (CSN) acts as a key regulatory complex that counteracts this process by removing NEDD8 from Cullin-3, thereby modulating its activity in a reversible manner (153).

#### 4.6.2 Other cholesterol transporters in tumor progression

Other ABC transporters also contribute to pediatric tumor progression. For instance, ABCG8, degraded by E3 ligases such as RNF5 and HRD1, correlates with metastasis in osteosarcoma, serving as a prognostic biomarker (154, 155). Conversely, ABCA6 exhibits tumor-suppressive properties in Ewing sarcoma, reducing intracellular cholesterol levels and inhibiting oncogenic pathways like IGF1R/AKT/MDM2. Enhancing ABCA6 expression improves chemosensitivity, particularly in combination with statins (156). Meanwhile, ABCB1 (P-glycoprotein) overexpression in NB promotes therapy resistance by effluxing cytotoxic drugs, with signaling pathways like Ras/ERK1/2/HIF-1α further exacerbating ABCA1 suppression and cholesterol dysregulation (145, 157).

### 4.7 Cholesterol esterification

Cholesterol esterification, mediated by ACAT1 and ACAT2, is essential for converting free cholesterol into cholesteryl esters, promoting lipid droplet formation and supporting tumor survival in cholesterol-rich environments. This metabolic adaptation drives tumor progression and therapy resistance in pediatric cancers.

ACAT1 plays a central role in NB by facilitating cholesterol esterification and lipid droplet accumulation. While this process supports tumor survival, it can induce oxidative stress and cell death under ER dysfunction. Silencing ACAT1 reduces COX2 expression and restores protective PKC/ERK signaling, highlighting its potential as a therapeutic target (158, 159). In glioblastoma, ACAT1 enhances cholesteryl ester synthesis to support membrane production and proliferation (160). Interestingly, ACAT1 inhibition also enhances the cytotoxic function of CD8(+) T cells, suggesting a potential immunomodulatory role in controlling tumor growth (161).

The UPS tightly controls ACAT enzyme stability, influencing cholesterol esterification and tumor metabolism. gp78 and INSIG mediate ROS-induced ubiquitination of ACAT2, promoting sterol storage and metabolic adaptation (161). In contrast, USP19 stabilizes ACAT1 by removing ubiquitin chains, enhancing cholesterol esterification and supporting tumor growth, as observed in hepatocellular carcinoma (162).

## 5 UPS-mediated regulation of FA in pediatric tumors

**Figure 2:**
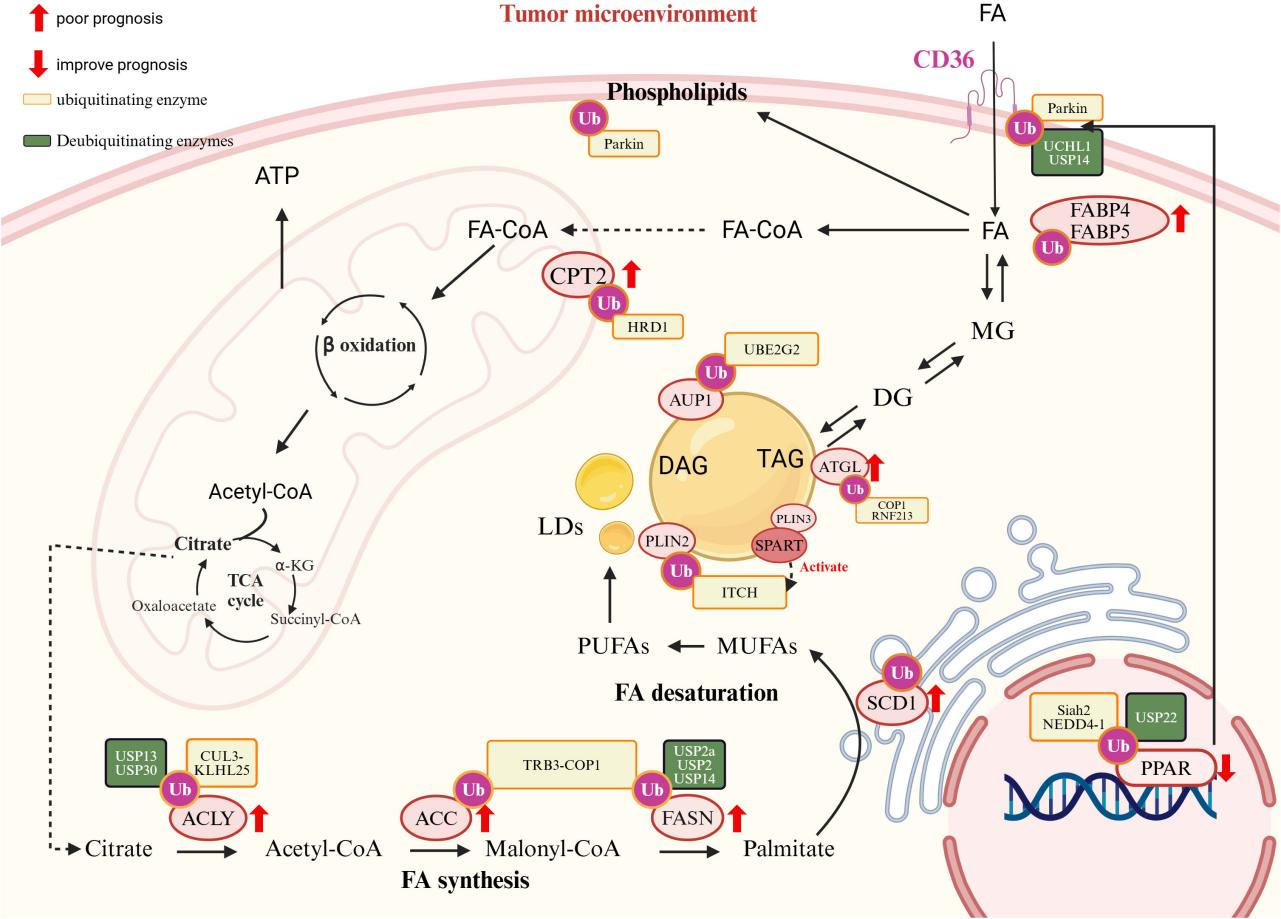
The role of ubiquitination in cholesterol metabolism and tumor prognosis. Cholesterol uptake: LDL receptor (LDLR) mediates cholesterol uptake. PCSK9 promotes LDLR degradation via the lysosome, while the E3 ubiquitin ligase IDOL ubiquitinates LDLR, targeting it for proteasomal degradation, reducing cholesterol uptake; Cholesterol Synthesis: The rate-limiting enzyme HMG-CoA reductase (HMGCR) is ubiquitinated by RNF145, gp78, HRD1, and RNF139, leading to degradation. Squalene epoxidase (SQLE) is ubiquitinated by MARCH6, affecting cholesterol biosynthesis; Cholesterol Efflux: ATP-binding cassette transporters ABCA1 and ABCG1 mediate cholesterol efflux. Their regulation involves ubiquitination by HUWE1, NEDD4-1, and others; Cholesterol Esterification: Acyl-CoA:cholesterol acyltransferase (ACAT) facilitates cholesterol storage in lipid droplets (LDs), regulated by gp78 and UPS19; Transcriptional Regulation: The sterol regulatory element-binding protein (SREBP) pathway controls cholesterol biosynthesis. SCAP facilitates SREBP transport from the ER to the Golgi, where it is activated. The INSIG-SCAP-SREBP complex is regulated via ubiquitination by gp78 and TRC8, while RNF145 and RNF5 ubiquitinate SCAP to modulate its stability. LXR activation induces genes like ABCA1, ABCG1, and IDOL, affecting cholesterol homeostasis. Cancer Metabolism and Prognostic Markers: EGFR signaling and glucose metabolism enhance SREBP activation, contributing to tumor lipid metabolism. Ubiquitination (orange boxes) and deubiquitination (green boxes) modulate key enzymes and pathways, influencing tumor progression and prognosis. Markers with red arrows (↑,↓) indicate prognostic impact, where upregulated elements are linked to poor prognosis, while downregulated components improve prognosis.

Fatty acids (FAs) are essential for membrane synthesis, energy production, and cellular signaling. Dysregulated FA metabolism in pediatric tumors supports proliferation, therapy resistance, and survival by balancing lipid storage and oxidative stress, such as lipid peroxidation (LPO) and ferroptosis (163). The UPS plays a central role in regulating key aspects of FA metabolism, including *de novo* synthesis, uptake, and β-oxidation (**Figure 2**).

### 5.1 Ubiquitination in *de novo* fatty acid synthesis

*De novo* fatty acid synthesis provides critical lipids for tumor membrane production and growth, with its key enzymes tightly regulated by the UPS.

ATP Citrate Lyase (ACLY), which links glucose metabolism to lipid synthesis, is ubiquitinated by the CUL3-KLHL25 E3 ligase, suppressing tumor growth by reducing lipid production (164). Conversely, deubiquitinases USP13 and USP30 stabilize ACLY, promoting tumor progression in cancers such as ovarian and liver cancers (165, 166). In osteosarcoma, ACLY drives tumor growth and metastasis via the XIST/miR-655/ACLY axis, while miR-22 downregulates ACLY to inhibit lipogenesis, highlighting its potential as a therapeutic target (167, 168).

Acetyl-CoA Carboxylase (ACC), a rate-limiting enzyme in FA synthesis, is ubiquitinated by COP1 under normal conditions to suppress lipogenesis (169). However, in certain cancers, ACC evades degradation through interactions with AKR1B10, enhancing FA synthesis and driving tumor progression (170). Additionally, AKR1B10 is strongly associated with chemotherapy resistance (171).

FASN, a key enzyme in lipogenesis, is upregulated in pediatric tumors such as osteosarcoma, NB, and medulloblastoma. In osteosarcoma, FASN promotes anoikis resistance and metastasis to the lungs (172, 173). In NB, FASN and SCD1 regulate unsaturated FA metabolism to enhance immune evasion and survival (173–176). Similarly, in medulloblastoma, FASN-driven lipid synthesis sustains tumor proliferation (177). The UPS critically regulates FASN. COP1 ubiquitinates FASN to prevent excessive lipid accumulation, while mutations in SPOP impair FASN degradation, promoting tumor growth (170). Deubiquitinases such as USP2a and USP14 stabilize FASN, enhancing lipogenesis and contributing to therapy resistance. For example, in retinoblastoma, USP14-mediated FASN stabilization suppresses ferroptosis and promotes resistance to cisplatin (61, 178, 179). Conversely, USP2a binds to and stabilizes MDM4, which in turn increases the mitochondrial localization of p53 and promotes apoptosis in glioma cells (180).

### 5.2 UPS-mediated control of fatty acid uptake and transport

Fatty acid (FA) uptake and transport are vital for tumor metabolism, influencing tumor cell proliferation, immune evasion, and the tumor microenvironment (TME). The UPS regulates key FA transporters, such as CD36 and fatty acid-binding proteins (FABPs), modulating lipid metabolism and tumor progression.

#### 5.2.1 CD36: a dual role in tumor metabolism and immunity

CD36, a key FA transporter, plays a complex role in tumor metabolism and immunity. Parkin-mediated mono-ubiquitination enhances CD36 activity, increasing FA uptake, while deubiquitinases like UCHL1 and USP14 stabilize CD36, preventing degradation (44, 181, 182). Dysregulated CD36 activity impacts both tumor growth and immune cell function.

In immune cells, CD36 overexpression impairs antitumor immunity. Excessive FA uptake in CD8+ T cells induces lipid peroxidation and ferroptosis, reducing cytotoxicity and immunotherapy efficacy (29). In TAMs and Tregs, CD36 promotes an immunosuppressive environment, supporting tumor growth. Targeting CD36 restores immune function and enhances antitumor responses (30, 183).

In pediatric tumors, CD36 has diverse effects. In osteosarcoma, CD36 drives proliferation and immune evasion through the CD36-MYD88 axis, correlating with poor prognosis. However, its interaction with Thrombospondin-1 (TSP-1) produces anti-angiogenic vasculostatin, suppressing tumor growth (184–186). In NB, CD36-mediated uptake of long-chain FAs like palmitic acid induces metabolic stress and cell damage, which can be alleviated by CD36 inhibition (187, 188). Beyond pediatric settings, CD36 enrichment in glioblastoma stem cells and other adult tumors associates with metastatic potential and poor outcomes (189, 190).

Fatty acid-binding proteins (FABPs) regulate intracellular FA transport and contribute to tumor progression. Although direct UPS regulation of FABPs is unclear, their roles in tumor metabolism and immune evasion are well-documented. FABP4 enhances pro-tumoral TAM phenotypes in NB, driving tumor progression. FABP4+ macrophage enrichment in metastatic osteosarcoma correlates with immune evasion and poor prognosis (191–193). FABP5 promotes growth in osteosarcoma through the Akt/mTOR pathway and mediates therapy resistance in medulloblastoma, underscoring its role in tumor survival and progression (194–196).

### 5.3 Ubiquitination in FAO

FAO is a crucial energy source for lipid-dependent pediatric tumors. The UPS regulates key enzymes in this pathway, influencing tumor progression, metastasis, and therapeutic outcomes.

Carnitine Palmitoyltransferase 1A (CPT1A), the rate-limiting enzyme in FAO, is regulated by UPS-mediated ubiquitination, which modulates its mitochondrial localization and activity. Elevated CPT1A expression promotes epithelial-mesenchymal transition (EMT), cancer stemness, and metastasis. In NB, high CPT1A levels correlate with poor prognosis, linking FAO activation to aggressive tumor behavior (21, 197). Conversely, CPT1B, a paralog of CPT1A, is associated with improved outcomes in osteosarcoma, highlighting isoform-specific roles in pediatric tumors (198).

CPT2, another key FAO enzyme, is regulated by the E3 ligase HRD1, which restricts FAO by targeting CPT2 for ubiquitination and degradation. Reduced HRD1 expression enhances CPT2 activity, driving FAO and tumorigenesis, as observed in triple-negative breast cancer (TNBC). Although direct evidence in pediatric tumors is limited, similar regulatory mechanisms are likely involved (199). Furthermore, Acyl-CoA Dehydrogenases (ACADs), key enzymes in the FAO pathway, are degraded by the UPS, restricting FAO activity and sensitizing tumor cells to energy deprivation. This regulation may present therapeutic opportunities by targeting energy metabolism in lipid-dependent tumors.

### 5.4 Cross-talk between ubiquitination and lipid droplets

Lipid droplets (LDs) act as dynamic energy reserves, releasing free fatty acids (FFAs) and cholesterol through cytosolic lipolysis or lipophagy to prevent lipotoxicity and support tumor metabolic demands. The UPS plays a crucial role in regulating LD turnover, enabling tumor cells to adapt to fluctuating energy needs.

The LD-associated protein AUP1 links LDs to the UPS by recruiting the E2 conjugase Ube2g2, facilitating ubiquitination on the LD surface (200). Coating proteins such as PLIN2 is ubiquitinated by the Itch E3 ligase, facilitating lipid droplet (LD) degradation and releasing free fatty acids (FFAs) to support tumor metabolism (201). SPART/SPG20 (Spartin) functions as an adaptor for Itch by binding to the LD-associated protein PLIN3, promoting the recruitment and activation of Itch. This leads to the ubiquitination of PLIN2 and the subsequent recruitment of the autophagy receptor SQSTM1/p62, triggering macrolipophagy (201, 202). Additionally, under nutrient stress, LDs can also undergo autophagy through ubiquitin-independent mechanisms (203).

Key enzymes regulating LD dynamics, such as ATGL and FSP27 (204), are also modulated by the UPS. For example, E3 ligases like COP1 and RNF213 ubiquitinate ATGL, reducing triglyceride hydrolysis and leading to increased LD accumulation. This shift not only augments the energy reserve of tumor cells but also enhances their metabolic flexibility—that is, the capacity to adjust fuel utilization and energy production in response to varying nutrient and stress conditions—which in turn supports tumor progression (205, 206). RNF213 suppresses carcinogenesis and affects MAPK/JNK signaling pathway in glioblastoma (207). These UPS-mediated mechanisms allow tumors to optimize energy utilization, contributing to their survival and growth under varying metabolic conditions (204, 208).

### 5.5 Transcriptional regulation of FA metabolism by UPS

The UPS regulates transcription factors such as PPARγ and SREBP1, which are central to fatty acid (FA) metabolism.

#### 5.5.1 Peroxisome proliferator-activated receptors (PPARs)

PPARγ, a nuclear receptor regulating FA metabolism and glucose homeostasis, plays dual roles in lipid metabolism and tumor biology. Highly expressed in pediatric cancers, PPARγ activation promotes tumor differentiation, inhibits proliferation, and induces apoptosis. For instance, PPARγ agonists enhance pro-apoptotic pathways (e.g., p21 and Bax), suppressing osteosarcoma growth (209, 210), and improve therapeutic responses in high-risk NB by promoting differentiation and metabolic reprogramming (211, 212). PPARγ is a critical regulator in tumor progression, and its expression is modulated by factors such as miR-27b and TRIM46, which in turn influence its impact on diseases like OS (213, 214).

The UPS provides post-translational control of PPARγ activity. Ligand-bound PPARγ undergoes K48-linked ubiquitination, limiting transcriptional activity (215). Under inflammatory conditions, E3 ligases such as Siah2 and NEDD4–1 mediate the ubiquitination and degradation of PPARγ. In contrast, deubiquitinases (DUBs) like USP22 remove K48-linked ubiquitin chains from PPARγ, stabilizing it. This stabilization enhances PPARγ’s transcriptional activity, leading to increased expression of lipogenic enzymes, including ACC and ACLY, which promote *de novo* fatty acid synthesis (216–218). Interestingly, PPARγ also acts as an E3 ligase, mediating ubiquitination at Lys28 and leading to p65 proteasomal degradation, directly linking lipid metabolism to immune modulation in the tumor microenvironment (219).Targeting the UPS-PPARγ axis holds potential for modulating FA metabolism and inflammation, particularly in pediatric cancers like osteosarcoma and NB (209–214, 220).

#### 5.5.2 Sterol regulatory element-binding protein 1 (SREBP1)

SREBP1 is a master regulator of lipogenic genes, driving FA synthesis to support tumor growth and metabolic adaptation. Although its role in pediatric cancers is underexplored, UPS-mediated regulation is essential for maintaining lipid homeostasis. The SCF-FBXW7 E3 ligase complex degrades SREBP1 via GSK3-dependent phosphorylation, restricting FA synthesis under metabolic stress (221). In contrast, another study demonstrates that SREBP2 targets FBXW7 to regulate miR-182, establishing a feedback loop that modulates SREBP transcriptional activity (222, 223). Conversely, SREBP1 acetylation stabilizes the protein by competing with ubiquitination, enhancing lipogenic activity (224). Other E3 ligases, such as RNF20 and RNF139, promote SREBP1 degradation or inhibit precursor synthesis under nutrient-limiting conditions (221, 225). Additionally, SIRT1-mediated deacetylation facilitates SREBP1 ubiquitination and degradation, linking lipid metabolism to cellular stress responses (226).

Upstream, mTORC1 integrates SREBP1 activation with tumor cell biosynthetic and energy demands, particularly under lipid-rich conditions, further enhancing SREBP1-driven FA synthesis to meet the needs of rapidly proliferating tumor cells (221, 227). We summarized lipid metabolism-related genes and their roles in tumor prognosis: regulation by the UPS in (**Table 1**).

**Table 1:** Lipid metabolism-related genes and their roles in tumor prognosis: regulation by the UPS.

Gene/ Protein	Ub/Dub Enzyme	Disease Relevance	Effect	Impact on lipid Metabolism/Other Functions	Notes	Reference
De Novo Synthesis						
***ACLY***	**CUL3-KLHL25**	OS, RMS, Various Cancers	poor prognosis	Regulates lipid deposition, β-catenin activation, and interacts with Wnt and mTOR pathways in tumor growth.	Therapeutic targeting of XIST/ACLY or miR-22 shows promise in reducing tumor progression; preclinical models suggest synergy with immune checkpoint inhibitors	(167, 168, 314, 315)
	**USP13, USP30**					
***FASN***	**COP1**	WT, OS, NB, MB, GC	Poor prognosis	Regulates lipid metabolism and tumor progression via HER2/PI3K/Akt, NFκB, and AMPK pathways. Promotes lipid accumulation, immune evasion, and unsaturated fatty acid synthesis.	Therapeutic targeting of FASN (e.g., Cerulenin, siFASN, miR-195, miR-424) reduces proliferation, metastasis, and invasion in various cancers. Combinatorial strategies improve outcomes by inhibiting Rb/AMPK signaling or reducing autophagy.	(174, 176, 177, 289, 316–321)
	**USP2a, USP2, USP14**					
***ACC***	COP1	Undefined	Poor prognosis	Ubiquitination by COP1 degrades ACC, inhibiting fatty acid synthesis. AKR1B10 stabilizes ACC, enhancing lipid synthesis and promoting tumor progression.	ACC degradation reduces lipid synthesis, providing a potential therapeutic target. AKR1B10 prevents ACC degradation, making it a target in lipid-driven cancers.	(169, 170)
	AKR1B10					
***SCD1***	Undefined	OS, HCC	Poor prognosis	Auto-protective role via PPARδ and Smad1/5 signaling in OS. ER stress induced by lipid desaturation suppresses MYCN in HCC, reducing proliferation.	Regulation under high shear force conditions in OS requires further examination. ACR reduces unsaturated fatty acids, inducing ER stress and MYCN inhibition in HCC.	(175, 322)
Fatty Acid Uptake and Transport						
***CD36***	**Parkin**	OS	Poor prognosis via inflammation; improved prognosis via anti-angiogenesis	High-risk ARGs (e.g., CBS, MYC, MMP3, CD36) promote tumor growth, immune evasion, and metastasis. TSP-1/CD36 axis inhibits angiogenesis and tumor progression	High-risk ARGs (e.g., CBS, CD36) correlate with poor survival in OS. Anti-CD36 therapies targeting TSP-1/CD36 loops reduce angiogenesis and tumor growth; identified drugs (e.g., Bortezomib, Sunitinib) show potential.	(184–187)
	**UCHL1, USP14**					
***FABP4***	Undefined	NB, OS	Poor prognosis	FABP4 binds ATPB, accelerating ubiquitination and reducing ATP levels in NB. In OS, FABP4 enhances lipid metabolism (FFAs, TGs) via the RPARP-AS1/Akt/mTOR pathway.	FABP4 promotes NB migration and invasion, suppressed by blocking IL1α. FABP4+ macrophages in metastatic OS are pro-inflammatory and linked to immunosuppressive microenvironments. Circulating FABP4 correlates with advanced stages.	(191, 194, 195)
***FABP5***	Undefined	NB, MB, OS	Poor prognosis	NBASP downregulates FABP5 in NB, inactivating MAPK and suppressing carcinogenesis. FABP5 enhances tumorigenic pathways (e.g., MYCN, E2F1, SREBF1/2) and lipid metabolism in NB and OS. Imbalanced CRABP-II↓/FABP5↑ leads to RA resistance in MB.	NBASP encoded by FAM201A is a potential therapeutic target. FABP5 knockdown reduces MYCN, survivin, and HMGA1 expression in NB. NSUN2-mediated m5C stabilizes FABP5 mRNA in OS, promoting fatty acid metabolism and tumor progression.	(192, 193, 196, 323)
Fatty Acid Oxidation						
***CPT1B***	Undefined	OS	improved prognosis	CPT1B expression correlates with immune cell regulation and better outcomes in OS.	SERPINE2 is linked to poor survival, while CPT1B suggests better prognosis. Biomarkers validated through AI and immunohistochemistry; potential for prognostic and therapeutic utility.	(198)
***CPT2***	**HRD1**	Undefined	Possibly Linked to Poor Prognosis	Undefined	Undefined	(199)
Lipid Droplets (LDs)						
***AUP1***	**UBE2G2**	Undefined	Undefined	Links lipid droplets (LDs) to UPS, mediating protein ubiquitination on the LD surface through Ube2g2.	AUP1 regulates LD turnover and protein quality control, suggesting a role in tumor metabolic adaptation and potential therapeutic targeting.	(200)
***PLIN2, PLIN5***	**Itch**	Undefined	Poor prognosis	PLIN2 and PLIN5 are ubiquitinated by Itch, linking lipid droplets (LDs) to the UPS for protein turnover and metabolic regulation.	Itch-mediated ubiquitination of PLIN2/PLIN5 regulates LD protein turnover, influencing tumor metabolic adaptation.	(202, 324)
***Spartin***	**Itch**	Undefined	Poor prognosis	Acts as an adaptor factor for autophagy-dependent lipid droplet (LD) turnover during nutrient deprivation.	Regulates LD degradation under metabolic stress, linking autophagy and lipid metabolism.	(202, 324)
***ATGL***	**COP1, RNF213**	Undefined	Undefined	Ubiquitination inhibits triglyceride hydrolysis, increasing lipid droplet (LD) content and disrupting lipid metabolism.		(205, 206)
***FSP27***	**COP1, RNF213**	Undefined	Undefined	UPS regulates the stability of FSP27, indirectly affecting lipid droplet homeostasis.		(204)
Transcriptional Regulation of Fatty Acid Metabolism						
***PPAR***	**Siah2, NEDD4-1, TRIM46**	MB, NB, OS	Improve prognosis	PPAR activation promotes tumor differentiation, apoptosis, and metabolic regulation. TRIM46-mediated PPAR ubiquitination enhances NF-κB signaling, promoting tumor growth and poor prognosis in OS.	PPAR agonists (e.g., fenofibrate, troglitazone) reduce tumor progression in MB, NB, and OS by modulating IGF-I, lipid metabolism, and pro-apoptotic pathways. TRIM46 regulates PPAR signaling, influencing tumor outcomes.	(211, 214, 220, 325–328)
	**USP22**					
***SREBP1***	**SCF-FBXW7, RNF20, RNF139, SIRT1,**	Undefined	poor prognosis	Ubiquitination (via SCF-FBXW7, RNF20, RNF139) or deacetylation (via SIRT1) promotes SREBP1 degradation, inhibiting fatty acid metabolism and maintaining lipid homeostasis. mTORC1 activation and SREBP1 acetylation enhance its stability and promote lipid metabolism, supporting tumor growth.	SCF-FBXW7, RNF20, and RNF139 promote SREBP1 degradation, reducing fatty acid synthesis. SIRT1 deacetylation links SREBP1 degradation to cellular stress responses. Acetylation or mTORC1 activation stabilizes SREBP1, driving tumor progression.	(213, 224, 226, 227)
Transcriptional Regulation of Cholesterol Metabolism						
***SREBP2***	**RNF145, SCF-FBXW7, TRC8**	NB	poor prognosis	Ubiquitination (via RNF145, SCF-FBXW7, TRC8) inhibits SREBP2 activation, reducing cholesterol synthesis and tumor growth. Deubiquitination by USP28 stabilizes SREBP2, enhancing cholesterol synthesis and lipid metabolism.	RNF145 and TRC8 inhibit SREBP2 activation via SCAP ubiquitination or ER-to-Golgi transport blockade. USP28 promotes tumor growth by stabilizing SREBP2, enhancing cholesterol metabolism.	(100–102, 106, 329)
	**USP28**					
***LXRs (LXRα, LXRβ)***	**IDOL, RNF145, SIRT1**	NB	Improve prognosis	24SOHC induces LXR-mediated adaptive responses; enhanced ABCG1 expression protects against 7KC-induced cytotoxicity; ABCA1 plays a less critical role in NB. 24(S)-hydroxycholesterol upregulates ApoE, ABCA1, and ABCG1 in astrocytes; NR Modulators inhibit mTOR signaling via DDIT4 in OS.	Adaptive responses enhanced by co-treatment with 9-cis retinoic acid; knockdown of LXR diminishes protective effects of 24SOHC. Statins show therapeutic potential; MES-NB addicted to mevalonate pathway.	(108)
Cholesterol uptake and LDLR (Low-Density Lipoprotein Receptor) pathway						
***LDLR***	**SREBP2, mTOR, IDOL, PCSK9**	NB, OS	poor prognosis	Activation of LXR induces IDOL, which ubiquitinates LDLR, promotes lysosomal degradation, and inhibits cholesterol uptake. During cholesterol deprivation, LDLR expression is induced, enhancing cholesterol uptake; PCSK9 binds to LDLR and directs it to lysosomal degradation, reducing LDLR levels on the cell surface and subsequently decreasing LDL uptake. mTOR activation can bypass the LXR-IDOL axis, maintaining high LDLR expression, thereby promoting cholesterol uptake and tumor metabolism.		(107–109, 118, 119, 330)
Cholesterol biosynthesis pathway						
***HMGCR***	**RNF145, gp78, HRD1, RNF139**	NB, RMS, MES-NB, OS	Poor Prognosis	Lovastatin modulates cholesterol biosynthesis and induces apoptosis in neuroblastoma; hyperactive Akt1 signaling promotes tumor progression in RMS; mesenchymal neuroblastomas depend on HMGCR-driven protein geranylgeranylation; inhibition of the mevalonate pathway reduces OS metastasis by suppressing Yes-associated protein 1 (YAP1) activity.	Statins exhibit therapeutic potential in neuroblastoma and osteosarcoma, reducing metastasis and tumor growth; combination therapy effective in RMS; mesenchymal subtype neuroblastomas show addiction to the mevalonate pathway.	(116, 122–126)
	**USP20**					
***SQLE*,** ***DHCR24, LDM***	**MARCHF6**	OS (High-Risk)	Poor Prognosis	Elevated SQLE promotes chemoresistance; suppression reduces malignancy, enhances chemosensitivity; regulates ferroptosis and cuproptosis.	SQLE is a metabolic vulnerability in OS; inhibitor FR194738 shows synergistic effects with chemotherapy.	(134, 141–143)
Cholesterol efflux						
***ABCA1***	**HUWE1, NEDD4-1, HECTD1,UBE3A, Cullin-3**	OS, MB, GBM, NB, Astrocytoma, AD	Poor prognosis in OS and GBM Protective role in NB and AD	Reduced cholesterol efflux impairs anti-tumor immune responses and promotes drug resistance in osteosarcoma and glioblastoma while enhanced efflux reduces oxidative stress in neuroblastoma and Alzheimer’s disease	Restoration of ABCA1 enhances chemo-immune sensitivity in osteosarcoma ABC inhibitors improve radiation sensitivity in medulloblastoma TP70 shows neuroprotective properties in Alzheimer’s disease	(145–147, 150)
***ABCG1***	**HUWE1, NEDD4-1, HECTD1**	NB, ES, OS	Poor/Protective Prognosis	Enhances cholesterol efflux and protects against oxidative damage in NB and astrocytoma. High expression in ES-CSCs and OST-CSCs confers drug resistance and promotes chemoresistance.	Targeting ABCG1 improves cholesterol efflux in neurodegenerative diseases and reduces chemoresistance in ES-CSCs and OST-CSCs.	(147–149)
***ABCA6***	Undefined	ES	Improve prognosis	Reduces intracellular cholesterol levels, inhibits IGF1R/AKT/MDM2 and IGF1R/AKT/mTOR signaling, increases chemosensitivity.	Acts as a tumor suppressor, enhancing statin efficacy and linking high expression to favorable outcomes in ES patients.	(156)
***ABCG8***	**RNF5, HRD1**	OS	poor prognosis	Identified as a hub gene associated with metastasis and survival; regulates osteosarcoma progression and metastatic potential.	Prognostic biomarker and therapeutic target; drugs targeting ABCG8 and co-regulated hub genes show potential for clinical application.	(154)
***ABCB1***	Undefined	NB	poor prognosis	Increased expression linked to drug resistance in OS, NB, and GBM; reduces intracellular drug accumulation, impairing treatment efficacy.	Promotes chemoresistance in OS via Ras/ERK1/2/HIF-1 axis; lapatinib inhibits efflux in NB to enhance YM155 cytotoxicity; associated with methylation-driven drug resistance in GBM.	(157)
Cholesterol esterification						
***ACAT1***	**USP19**	NB, GBM	poor prognosis	Promotes cholesteryl ester formation in GBM and 24S-OHC-induced neurotoxicity in NB.	Excessive 24S-OHC esterification in NB causes neuronal death; ACAT1 activity drives cholesteryl ester biosynthesis in GBM.	(158)
***ACAT2***	**gp78**	Undefined	poor prognosis	ROS-induced ubiquitination of ACAT2 enhances its degradation, promoting cholesterol storage and metabolic adaptation.		(161)

Tumor types – OS, osteosarcoma; RMS, rhabdomyosarcoma; NB, neuroblastoma; MB, medulloblastoma; GC, gastric cancer; HCC, hepatocellular carcinoma; WT, wild type; GBM, glioblastoma; ES, Ewing sarcoma; AD, Alzheimer’s disease; Astrocytoma.

Enzymes/Proteins – ACLY, ATP citrate lyase; FASN, fatty acid synthase; ACC, acetyl‐CoA carboxylase; SCD1, stearoyl‐CoA desaturase 1; CD36, FABP4/5, fatty acid-binding protein 4/5; CPT1B/CPT2, carnitine palmitoyltransferase 1B/2; AUP1, PLIN2/5, Spartin, ATGL, FSP27.

Transcriptional Regulators – PPAR, peroxisome proliferator-activated receptor; SREBP1/2, sterol regulatory element-binding protein 1/2; HMGCR, 3-hydroxy-3-methylglutaryl-CoA reductase; YAP1 denotes Yes-associated protein 1.

Cholesterol Metabolism Components – IDOL, inducible degrader of the LDL receptor; PCSK9, proprotein convertase subtilisin/kexin type 9; ABCA1/ABCG1/ABCA6/ABCG8/ABCB1, ATP-binding cassette transporters; ACAT1/2, acyl-CoA cholesterol acyltransferase 1/2.

Ubiquitination/Deubiquitination Enzymes – CUL3-KLHL25, USP13, USP30, COP1, USP2a/USP2, AKR1B10, HRD1, UBE2G2, Itch, RNF213, Siah2, NEDD4-1, TRIM46, USP22, SCF-FBXW7, RNF20, RNF139, SIRT1, RNF145, TRC8, USP28, gp78, USP20, MARCHF6, HUWE1, NEDD4-1, HECTD1, UBE3A, Cullin-3, RNF5.

Others – XIST, miR-22, miR-195, miR-424, NBASP, CRABP-II, MAPK, MYCN, E2F1, SREBF1/2, ER, ACR, ATPB, TSP-1, RPARP-AS1, mTOR, NFκB, AMPK, HER2, PI3K/Akt, Rb, SERPINE2, DDIT4, 24SOHC, 7KC, ApoE, IGF-I, MDM2.

## 6 Ubiquitin ligases indirectly influence tumor lipid reprogramming

E3 ubiquitin ligases play critical roles in tumor lipid reprogramming by targeting regulatory proteins for degradation, driving tumor progression and therapy resistance. In Group 3 medulloblastoma, RNF126 ubiquitinates FSP1, altering its plasma membrane localization and disrupting the CoQ/CoQH2 balance, thereby suppressing ferroptosis and preventing phospholipid peroxidation. RNF126 overexpression correlates with poor prognosis in this subtype (228). In triple-negative breast cancer(TNBC), depletion of RNF126 increased sensitivity to irradiation (229). Similarly, in NB, TRIM59 promotes chemoresistance and tumor growth by degrading p53 and suppressing ferroptosis, enabling tumor survival under stress (230).

FBW7 regulates lipid metabolism by targeting c-Myc for degradation. Loss of FBW7 stabilizes c-Myc, resulting in abnormal lipid accumulation, uncontrolled proliferation, and therapy resistance (231). In osteosarcoma, FBW7 downregulation reduces the degradation of KCa1.1, a potassium channel localized in lipid rafts, which enhances drug resistance. Pharmacological inhibition of KCa1.1 restores chemosensitivity, demonstrating its therapeutic potential (232).

By modulating ferroptosis, lipid raft dynamics, and energy metabolism, E3 ligases such as RNF126, TRIM59, and FBW7 enable tumors to adapt to metabolic stress and evade apoptosis, making them promising targets for disrupting tumor lipid reprogramming.

## 7 Lipid regulation of ubiquitination in tumor progression and resistance

Lipids, categorized into eight classes by the LIPID MAPS system, are essential for cellular metabolism and signaling (233). Their interplay with ubiquitination reprograms tumor lipid metabolism, influencing key protein degradation and regulation. Imbalances in lipid ratios and dysregulated lipid metabolism-related genes drive abnormal lipid synthesis, uncontrolled cell growth, and tumor progression.

In Ewing sarcoma, ceramide triggers cleavage of the intracellular domain of GPR64, which translocates to the nucleus and promotes ubiquitination of RIF1 through the Cullin3-RING E3 ligase complex and SPOP, driving tumor growth (234). Similarly, lyso-Gb3, a deacylated lipid derivative, enhances ubiquitination of molecular chaperones like HSP90 and HSP60, inducing cytotoxicity and disrupting protein homeostasis (235). In osteosarcoma, M2 macrophage-derived exosomal Apoc1 facilitates ACSF2 ubiquitination and degradation, suppressing ferroptosis and promoting tumor progression (236).

Lipid metabolites also contribute to therapeutic resistance. For example, Prostaglandin E1 (PGE1) activates cAMP-PKA signaling via the EP4 receptor, promoting GLI2 ubiquitination and degradation to suppress Hedgehog signaling, overcoming GLI2 amplification-associated drug resistance in medulloblastoma models (237). Additionally, FASN modulates cell cycle regulation by stabilizing p27^Kip1 through Skp2 suppression, linking lipid metabolism to tumor cell cycle arrest (238). Mitochondrial lipids like cardiolipin and phosphatidic acid regulate mitochondrial dynamics via MARCH5 ubiquitination. Cardiolipin accelerates MARCH5 self-ubiquitination, promoting mitochondrial turnover, while phosphatidic acid stabilizes MARCH5 to preserve mitochondrial homeostasis (239). Furthermore, PD-1 signaling facilitates MARCH5-mediated γ-chain ubiquitination, impairing antitumor immunity. Combining PD-1 blockade with MARCH5 inhibitors and IL-2 enhances immune responses and therapeutic efficacy (240).

A recent study by Sakamaki et al. demonstrated that phospholipids, such as phosphatidylethanolamine (PE), can be ubiquitinated in endosomal and lysosomal compartments by the ubiquitin enzymes Uba1 (E1), Ubc4/5 (E2), and Tul1 (E3). These findings provide insights into the novel roles of lipids beyond metabolic substrates (241). This reciprocal interaction between lipid metabolism and ubiquitination offers novel insights into tumor biology and identifies potential therapeutic targets.

## 8 Therapeutic strategies and discussion: targeting the UPS in pediatric tumors

Current research has demonstrated that the UPS plays a crucial role in the regulation of pediatric solid tumors, including NB (242). The ubiquitination pathway has been explored for drug development in various diseases, including cancer (243–246). 这However, directly targeting E3 ligases is challenging due to their multiple substrates, which may lead to unintended effects. A more effective approach could be inhibiting downstream pathways or specific protein-protein interactions (PPIs) relevant to cancer.

Currently, drug development targeting the ubiquitination pathway focuses on three main strategies:

Inhibiting proteasome-mediated proteolysis to disrupt protein degradation and affect tumor cell homeostasis.
Targeted protein degradation (TPD) using techniques like PROTACs to selectively degrade oncogenic proteins.
Inhibiting key factors, particularly E3 ligases, to regulate tumor progression.

The ubiquitination pathway is highly complex and can have oncogenic or tumor-suppressive effects depending on the specific context (243, 244, 247, 248). Notably, both E3 ligases and deubiquitinating enzymes (DUBs) play key roles in lipid biosynthesis and degradation, processes closely linked to tumor progression. However, a comprehensive understanding of the clinical relevance of these lipid metabolism-related UPS components in pediatric tumors is still lacking.

The following sections summarize current strategies, preclinical findings, and future perspectives.

### 8.1 Proteasome inhibitors

Bortezomib—a proteasome inhibitor approved by the FDA for multiple myeloma and mantle cell lymphoma—has demonstrated promising preclinical activity in pediatric tumor models (249). In animal studies, bortezomib not only suppressed the growth of NB cells injected into mice (249) but also induced apoptosis in human medulloblastoma cells by inhibiting key signaling pathways, including AKT and NF−κB (250). Based on both *in vitro* experiments and mouse xenograft models, Yang et al. proposed that bortezomib might be effective for treating pediatric MBs (250). Additionally, bortezomib effectively inhibits NB cell growth and angiogenesis (251). However, its limited ability to cross the blood–brain barrier may restrict its efficacy in treating central nervous system (CNS) tumors (252). In the clinical setting, besides bortezomib (252–254), other FDA-approved proteasome inhibitors such as carfilzomib (255, 256) and ixazomib (257, 258) are currently used to treat myeloma (248, 259, 260).

The effects of proteasome inhibitors on apoptosis are multifaceted and depend heavily on the cellular context. The UPS governs a variety of proteins involved in programmed cell death. For example, in HL60 leukemia cells, these inhibitors promote apoptosis (261), yet in sympathetic neurons, they might have a protective, anti-apoptotic effect (262). Research by Bobba et al. demonstrated that in 7-day-old rat cerebellar granule neurons, proteasome inhibition can prevent the release of cytochrome c during apoptosis, suggesting that normal proteasomal function is crucial for initiating cell death and that its inhibition may rescue cells from dying (263). Complementary work by Butts et al. revealed a dual-phase response in 7- to 8-day-old rat cerebellar granule cells: brief inhibition boosts cell survival by upregulating factors like MEF2D, whereas extended inhibition elevates pro-death molecules such as c-JUN and Bim, ultimately causing toxicity (264). These results emphasize that the duration and concentration of proteasome inhibitor exposure are key factors in determining whether the response will be protective or deleterious, with malignant cells generally undergoing apoptosis in response (264–266).

### 8.2 Deubiquitination module

#### 8.2.1 USP22

USP22 plays a pivotal role in tumor cell tolerance to chemotherapy by promoting the repair of DNA strand breaks (267). In osteosarcoma, silencing ALKBH5 increases m6A modifications that destabilize USP22 and RNF40, leading to the downregulation of genes involved in the cell cycle, DNA replication, and repair, whereas enhanced ALKBH5 activity drives USP22/RNF40-dependent oncogenic processes (268).Moreover, USP22 contributes to osteosarcoma progression by modulating glycolytic pathways that affect cell proliferation, apoptosis, migration, and invasion (269). In hepatocellular carcinoma, USP22 knockdown or inhibition via agents like SO_2_ markedly increases the sensitivity of HCC LR and primary HCC cells to lenvatinib, while also diminishing Treg-mediated immunosuppression and enhancing CD8^+^ T cell infiltration, suggesting a promising role in cancer immunotherapy (270, 271). Similarly, in non-small cell lung cancer, USP22 inhibition improves gefitinib sensitivity and induces ferroptosis, offering a novel strategy to overcome chemoresistance (272).

Structure-based virtual screening has identified selective USP22 inhibitors such as rottlerin and morusin, which elevate histone monoubiquitination and lower Sirt1 and PD-L1 expression, thereby exhibiting significant antitumor activity *in vivo*(273). Additionally, overexpression of miR-200b-5p, a regulator of USP22, suppresses gastric cancer cell proliferation and migration by targeting the NF−κB pathway, highlighting its potential as a new therapeutic target in gastric cancer (274).

#### 8.2.2 USP13

Recent studies highlight USP13’s critical role in tumor progression. In osteosarcoma, Spautin-1–mediated inhibition of USP13 degrades METTL3, destabilizing ATG5 mRNA and impairing autophagy and glycolysis, which ultimately suppresses tumor growth (275). In glioblastoma, both Spautin-1 treatment and USP10/USP13 knockdown reduce cell proliferation and migration (276). Notably, high USP13 expression in glioma stem cells stabilizes c-Myc, while its depletion enhances c-Myc ubiquitination and degradation, effectively inhibiting tumor growth; conversely, overexpression of FBXL14 promotes c-Myc degradation, leading to stem cell differentiation and further tumor suppression (277).

#### 8.2.3 USP30

Biochemical and structural studies have identified selective inhibitors for USP30. For instance, the benzosulfonamide compound USP30inh shows high selectivity and potency for USP30 over 49 other deubiquitylating enzymes in a NB cell line (278). Similarly, the N−cyano pyrrolidine FT3967385 is a highly selective USP30 inhibitor that accelerates the PINK1−dependent generation of phospho−Ser65 ubiquitin in cells (279). In parallel, the long non−coding RNA USP30−AS1 has emerged as a valuable prognostic marker; high expression of USP30−AS1 is associated with poor survival in both primary and recurrent glioma patients, likely due to its repression of mitophagy and disruption of mitochondrial homeostasis (280). Moreover, USP30−AS1 is currently under investigation as a potential biomarker for osteosarcoma prognosis (281).

#### 8.2.4 USP2

USP2 is a deubiquitinating enzyme that modulates key signaling pathways by regulating protein stability. It stabilizes β−catenin, thereby promoting epithelial-to-mesenchymal transition (EMT) and altering chemosensitivity (282). In sorafenib−resistant Huf7−SR cells, USP2 inhibition reduces cFILP expression, induces apoptosis, and enhances sorafenib sensitivity (283), whereas USP2 overexpression in renal carcinoma cells (A498 and CAKi−1) suppresses proliferation, migration, and invasion (284).Therapeutically, USP2 is an attractive target; ML364 is the most extensively studied USP2 inhibitor, with other inhibitors like Q29, STD1T, and LCAHA under investigation (285). In mantle cell lymphoma, ML364 facilitates the degradation of CCND1 and β−catenin, arresting the cell cycle and impeding tumor growth (286). Moreover, agents such as 6−thioguanine (6−TG) are clinically employed for AML and CML (287, 288). Additionally, USP2, recruited by VCP, enhances the stability of fatty acid synthase (FASN) by removing K48−linked ubiquitin chains, a process whose inhibition reverses pro-tumorigenic effects in osteosarcoma cells (289). Elevated USP2 expression is also associated with poor prognosis in medulloblastoma (290).

#### 8.2.5 USP28

Additionally, inhibitors of USP25/28 (e.g., CT1113) effectively lower MYCN expression in NB, as confirmed in patient-derived xenograft models (291). USP28 is similarly overexpressed in glioblastoma, contributing to tumorigenicity through MYC regulation (292).

### 8.3 Ubiquitination module

#### 8.3.1 ITCH

ITCH, a HECT-type E3 ubiquitin ligase, plays critical roles in regulating apoptosis, cell growth, and inflammation. Dysregulated ITCH expression can impair the apoptotic response to conventional chemotherapy (293–295). In NB, ITCH downregulates TAp73 levels, thereby contributing to chemoresistance (296). Desmethyl-clomipramine (DCMI), the active metabolite of clomipramine, inhibits both ITCH autoubiquitylation and its ubiquitylation of p73, enhancing the cytotoxic effects of standard chemotherapeutics in various cancers (297, 298) In glioblastoma, gain-of-function TP53 mutations in astrocytes upregulate ITCH, leading to ferritin heavy chain (FTH) degradation and increased free iron levels (299). Conversely, in NB, ITCH cooperates with UBE4B to promote the polyubiquitination of Ku70 and c-FLIPL, thereby enhancing apoptosis (300). Additionally, nanoparticle-mediated silencing of ITCH sensitizes tumor cells to irradiation, offering a potential strategy for combined chemo- and radiotherapy, particularly in p53-deficient tumors (301).

#### 8.3.2 NEDD4

I3C, a natural inhibitor derived from Brassicaceae, effectively targets NEDD4 and WWP1, and has been shown to inhibit NEDD4–1 activity (302). In temozolomide (TMZ)-resistant glioblastoma cells, NEDD4–1 contributes to redox imbalance by promoting PTEN degradation and activating the AKT/NRF2/HO-1 pathway, while I3C treatment suppresses tumorsphere formation, migration, and invasion in resistant cell lines (303).

#### 8.3.3 FBXW7

FBXW7 is a key tumor suppressor whose dysregulation contributes to malignant progression across multiple cancer types. In Ewing sarcoma, for instance, KDM5B attenuates FBXW7 transcription, leading to CCNE1 accumulation and enhanced proliferation (304). Similarly, in glioblastoma, miR-92b targets FBXW7 and TRIP13 suppresses its transcription, thereby stabilizing oncogenic proteins like c−MYC and driving tumorigenesis (305, 306). In osteosarcoma, the lncRNA GClnc1 facilitates tumor progression by stabilizing NONO and preventing FBXW7-mediated ubiquitination (307), whereas targeting miR-27a−3p can restore FBXW7 expression to sensitize Taxol−resistant cells (308). Exosomal miR-25−3p in glioblastoma also promotes proliferation and temozolomide resistance by targeting FBXW7 (309).

Our own findings further support the pivotal role of FBXW7. Agents such as IPA and N6−BA modulate GBM cell proliferation by regulating the turnover of key proteins—like SREBPs and Mcl1—thereby influencing malignant progression and chemoresistance (306). In MYC−driven medulloblastoma, inhibition of PLK1 stabilizes FBXW7, underscoring its tumor‐suppressive function, while blocking ELK1−mediated transcription activates FBXW7 and suppresses MCL1, leading to cytotoxicity (310) (311). In the context of MYCN−amplified neuroblastoma, Aurora−A inhibitors (e.g., MLN8054 and MLN8237) disrupt the protective Aurora−A/N−Myc complex, promoting FBXW7−mediated degradation of N−Myc and thus inhibiting tumor growth (291).

Moreover, insights from yeast have provided a structural basis for targeting this pathway. SCF−I2, an inhibitor of the yeast F−box protein Cdc4 (the homolog of human FBXW7), binds to the Skp1–Cdc4 complex through an allosteric mechanism—a mode of action distinct from inhibitors like nutlin, which block protein–protein interactions directly (PDB: 3MKS) (312).

#### 8.3.4 RNF5

High RNF5 expression in neuroblastoma and melanoma is associated with improved patient survival, suggesting a tumor-suppressive role. Activation of RNF5 with the agonist Analog-1 reduces cell viability by inhibiting F_1_F_0_ ATPase activity (313). This inhibition limits glutamine supply and elevates oxidative stress, together delaying tumor growth (313).

### 8.4 Future challenges and innovative directions

#### 8.4.1 Mechanistic complexity

A deeper, context-specific understanding of the UPS’s regulatory networks in pediatric tumors is essential. Future research must elucidate how distinct E3 ligases and deubiquitinases coordinate to regulate lipid metabolic pathways, thereby offering insight into novel, age-specific vulnerabilities.

#### 8.4.2 Selective targeting and drug development

Given the complex, large-scale interactions involved in substrate–E3 binding, the development of small-molecule inhibitors that are both selective and minimally toxic remains a formidable challenge. Innovative strategies—such as leveraging high-throughput screening and structure-based drug design—are needed to identify agents that precisely modulate these interactions.

#### 8.4.3 Resistance mechanisms and combination therapies

Tumor cells may develop resistance through adaptive metabolic rewiring and alterations in UPS regulation. Addressing this requires exploring combination therapies that integrate UPS inhibitors with established modalities (chemotherapy, radiotherapy, or immunotherapy) to preempt and overcome resistance.

#### 8.4.4 Age-specific therapeutic strategies

The distinct metabolic and developmental characteristics of pediatric tumors call for tailored therapeutic approaches. Future clinical strategies must account for these differences, optimizing dosing and safety profiles to ensure effective, child-specific treatments.

#### 8.4.5 Integration of immune modulation

As lipid metabolism also shapes the tumor immune microenvironment, future research should investigate how targeting the UPS could concurrently modulate immune responses. This dual approach might not only impede tumor growth but also enhance immunotherapeutic efficacy, paving the way for more comprehensive treatment regimens.

In summary, while the UPS-driven regulation of lipid metabolism presents a promising frontier in pediatric oncology, realizing its full therapeutic potential will require overcoming significant mechanistic and translational challenges. Advancing our understanding in these areas promises to yield innovative, targeted strategies that can more effectively disrupt tumor metabolism and improve outcomes for pediatric cancer patients.

## References

[1] Wang S Jiang Y Zheng R Yu Y Wei W Li N. Mobilizing China and the global community to confront the treatment desert for pediatric solid tumors. Cancer Discov. (2024) 14:26–9. doi: 10.1158/2159-8290.CD-23-0451 PMC10784741 38213295

[2] Mattano LA Jr. Devidas M Maloney KW Wang C Friedmann AM Buckley P. Favorable trisomies and ETV6-RUNX1 predict cure in low-risk B-cell acute lymphoblastic leukemia: results from children's oncology group trial AALL0331. J Clin Oncol. (2021) 39:1540–52. doi: 10.1200/JCO.20.02370 PMC8274747 33739852

[3] Ward E DeSantis C Robbins A Kohler B Jemal A. Childhood and adolescent cancer statistics, 2014. CA Cancer J Clin. (2014) 64:83–103. doi: 10.3322/caac.21219 24488779

[4] Ni X Li Z Li X Zhang X Bai G Liu Y. Socioeconomic inequalities in cancer incidence and access to health services among children and adolescents in China: a cross-sectional study. Lancet. (2022) 400:1020–32. doi: 10.1016/S0140-6736(22)01541-0 36154677

[5] Tutelman PR Heathcote LC. Fear of cancer recurrence in childhood cancer survivors: A developmental perspective from infancy to young adulthood. Psychooncology. (2020) 29:1959–67. doi: 10.1002/pon.v29.11 33068463

[6] Sun CX Daniel P Bradshaw G Shi H Loi M Chew N. Generation and multi-dimensional profiling of a childhood cancer cell line atlas defines new therapeutic opportunities. Cancer Cell. (2023) 41:660–677.e7. doi: 10.1016/j.ccell.2023.03.007 37001527

[7] Bacci M Lorito N Smiriglia A Morandi A. Fat and furious: lipid metabolism in antitumoral therapy response and resistance. Trends Cancer. (2021) 7:198–213. doi: 10.1016/j.trecan.2020.10.004 33281098

[8] Yang F Hu A Guo Y Wang J Li D Wang X. p113 isoform encoded by CUX1 circular RNA drives tumor progression via facilitating ZRF1/BRD4 transactivation. Mol Cancer. (2021) 20:123. doi: 10.1186/s12943-021-01421-8 34579723 PMC8474885

[9] Budhu A Roessler S Zhao X Yu Z Forgues M Ji J. Integrated metabolite and gene expression profiles identify lipid biomarkers associated with progression of hepatocellular carcinoma and patient outcomes. Gastroenterology. (2013) 144:1066–1075.e1. doi: 10.1053/j.gastro.2013.01.054 23376425 PMC3633738

[10] Zaugg K Yao Y Reilly PT Kannan K Kiarash R Mason J. Carnitine palmitoyltransferase 1C promotes cell survival and tumor growth under conditions of metabolic stress. Genes Dev. (2011) 25:1041–51. doi: 10.1101/gad.1987211 PMC3093120 21576264

[11] Cheng C Geng F Cheng X Guo D. Lipid metabolism reprogramming and its potential targets in cancer. Cancer Commun (Lond). (2018) 38:27. doi: 10.1186/s40880-018-0301-4 29784041 PMC5993136

[12] Menendez JA Lupu R. Fatty acid synthase and the lipogenic phenotype in cancer pathogenesis. Nat Rev Cancer. (2007) 7:763–77. doi: 10.1038/nrc2222 17882277

[13] Röhrig F Schulze A. The multifaceted roles of fatty acid synthesis in cancer. Nat Rev Cancer. (2016) 16:732–49. doi: 10.1038/nrc.2016.89 27658529

[14] Yang K Wang X Song C He Z Wang R Xu Y. The role of lipid metabolic reprogramming in tumor microenvironment. Theranostics. (2023) 13:1774–808. doi: 10.7150/thno.82920 PMC10091885 37064872

[15] Bian X Liu R Meng Y Xing D Xu D Lu Z. Lipid metabolism and cancer. J Exp Med. (2021) 218. doi: 10.1084/jem.20201606 PMC7754673 33601415

[16] Caro P Kishan AU Norberg E Stanley IA Chapuy B Ficarro SB. Metabolic signatures uncover distinct targets in molecular subsets of diffuse large B cell lymphoma. Cancer Cell. (2012) 22:547–60. doi: 10.1016/j.ccr.2012.08.014 PMC3479446 23079663

[17] Ruiz-Pérez MV Sainero-Alcolado L Oliynyk G Matuschek I Balboni N Ubhayasekera S. Inhibition of fatty acid synthesis induces differentiation and reduces tumor burden in childhood neuroblastoma. iScience. (2021) 24:102128. doi: 10.1016/j.isci.2021.102128 33659885 PMC7895756

[18] Merino Salvador M Gómez de Cedrón M Moreno Rubio J Falagán Martínez S Sánchez Martínez R Casado E. Lipid metabolism and lung cancer. Crit Rev Oncol Hematol. (2017) 112:31–40. doi: 10.1016/j.critrevonc.2017.02.001 28325263

[19] Zipinotti Dos Santos D de Souza JC Pimenta TM da Silva Martins B Junior RSR Butzene SMS. The impact of lipid metabolism on breast cancer: a review about its role in tumorigenesis and immune escape. Cell Commun Signal. (2023) 21:161. doi: 10.1186/s12964-023-01178-1 37370164 PMC10304265

[20] Alannan M Fayyad-Kazan H Trézéguet V Merched A. Targeting lipid metabolism in liver cancer. Biochemistry. (2020) 59:3951–64. doi: 10.1021/acs.biochem.0c00477 32930581

[21] Agostini M Melino G Habeb B Calandria JM Bazan NG. Targeting lipid metabolism in cancer: neuroblastoma. Cancer Metastasis Rev. (2022) 41:255–60. doi: 10.1007/s10555-022-10040-8 PMC9363363 35687185

[22] Drieu A Du S Storck SE Rustenhoven J Papadopoulos Z Dykstra T. Parenchymal border macrophages regulate the flow dynamics of the cerebrospinal fluid. Nature. (2022) 611:585–93. doi: 10.1038/s41586-022-05397-3 PMC9899827 36352225

[23] Shapouri-Moghaddam A Mohammadian S Vazini H Taghadosi M Esmaeili SA Mardani F. Macrophage plasticity, polarization, and function in health and disease. J Cell Physiol. (2018) 233:6425–40. doi: 10.1002/jcp.v233.9 PMC13482560 29319160

[24] Biswas SK Mantovani A. Macrophage plasticity and interaction with lymphocyte subsets: cancer as a paradigm. Nat Immunol. (2010) 11:889–96. doi: 10.1038/ni.1937 20856220

[25] Mills CD. M1 and M2 macrophages: oracles of health and disease. Crit Rev Immunol. (2012) 32:463–88. doi: 10.1615/CritRevImmunol.v32.i6.10 23428224

[26] Noy R Pollard JW. Tumor-associated macrophages: from mechanisms to therapy. Immunity. (2014) 41:49–61. doi: 10.1016/j.immuni.2014.06.010 25035953 PMC4137410

[27] Yuan A Hsiao YJ Chen HY Chen HW Ho CC Chen YY. Opposite effects of M1 and M2 macrophage subtypes on lung cancer progression. Sci Rep. (2015) 5:14273. doi: 10.1038/srep14273 26399191 PMC4585843

[28] Pearce EL Pearce EJ. Metabolic pathways in immune cell activation and quiescence. Immunity. (2013) 38:633–43. doi: 10.1016/j.immuni.2013.04.005 PMC3654249 23601682

[29] Ma X Xiao L Liu L Ye L Su P Bi E. CD36-mediated ferroptosis dampens intratumoral CD8(+) T cell effector function and impairs their antitumor ability. Cell Metab. (2021) 33:1001–1012.e5. doi: 10.1016/j.cmet.2021.02.015 33691090 PMC8102368

[30] Xu S Chaudhary O Rodríguez-Morales P Sun X Chen D Zappasodi R. Uptake of oxidized lipids by the scavenger receptor CD36 promotes lipid peroxidation and dysfunction in CD8(+) T cells in tumors. Immunity. (2021) 54:1561–1577.e7. doi: 10.1016/j.immuni.2021.05.003 34102100 PMC9273026

[31] Ma X Bi E Lu Y Su P Huang C Liu L. Cholesterol induces CD8(+) T cell exhaustion in the tumor microenvironment. Cell Metab. (2019) 30:143–156.e5. doi: 10.1016/j.cmet.2019.04.002 31031094 PMC7061417

[32] Wu H Han Y Rodriguez Sillke Y Deng H Siddiqui S Treese C. Lipid droplet-dependent fatty acid metabolism controls the immune suppressive phenotype of tumor-associated macrophages. EMBO Mol Med. (2019) 11:e10698. doi: 10.15252/emmm.201910698 31602788 PMC6835560

[33] Wu L Zhang X Zheng L Zhao H Yan G Zhang Q. RIPK3 orchestrates fatty acid metabolism in tumor-associated macrophages and hepatocarcinogenesis. Cancer Immunol Res. (2020) 8:710–21. doi: 10.1158/2326-6066.CIR-19-0261 32122992

[34] Hou Y Wei D Zhang Z Guo H Li S Zhang J. FABP5 controls macrophage alternative activation and allergic asthma by selectively programming long-chain unsaturated fatty acid metabolism. Cell Rep. (2022) 41:111668. doi: 10.1016/j.celrep.2022.111668 36384126

[35] Dirat B Bochet L Dabek M Daviaud D Dauvillier S Majed B. Cancer-associated adipocytes exhibit an activated phenotype and contribute to breast cancer invasion. Cancer Res. (2011) 71:2455–65. doi: 10.1158/0008-5472.CAN-10-3323 21459803

[36] Almeida L Dhillon-LaBrooy A Carriche G Berod L Sparwasser T. CD4(+) T-cell differentiation and function: Unifying glycolysis, fatty acid oxidation, polyamines NAD mitochondria. J Allergy Clin Immunol. (2021) 148:16–32. doi: 10.1016/j.jaci.2021.03.033 33966898

[37] Ladanyi A Mukherjee A Kenny HA Johnson A Mitra AK Sundaresan S. Adipocyte-induced CD36 expression drives ovarian cancer progression and metastasis. Oncogene. (2018) 37:2285–301. doi: 10.1038/s41388-017-0093-z PMC5920730 29398710

[38] Singh SV Ajay AK Mohammad N Malvi P Chaube B Meena AS. Proteasomal inhibition sensitizes cervical cancer cells to mitomycin C-induced bystander effect: the role of tumor microenvironment. Cell Death Dis. (2015) 6:e1934. doi: 10.1038/cddis.2015.292 26492368 PMC4632313

[39] Vergani E Beretta GL Aloisi M Costantino M Corno C Frigerio S. Targeting of the lipid metabolism impairs resistance to BRAF kinase inhibitor in melanoma. Front Cell Dev Biol. (2022) 10:927118. doi: 10.3389/fcell.2022.927118 35912092 PMC9326082

[40] Loix M Zelcer N Bogie JFJ Hendriks JJA. The ubiquitous role of ubiquitination in lipid metabolism. Trends Cell Biol. (2024) 34:416–29. doi: 10.1016/j.tcb.2023.09.001 37770289

[41] Antao AM Tyagi A Kim KS Ramakrishna S. Advances in deubiquitinating enzyme inhibition and applications in cancer therapeutics. Cancers (Basel). (2020) 12. doi: 10.3390/cancers12061579 PMC7352412 32549302

[42] Mani A Gelmann EP. The ubiquitin-proteasome pathway and its role in cancer. J Clin Oncol. (2005) 23:4776–89. doi: 10.1200/JCO.2005.05.081 16034054

[43] Zheng Q Huang T Zhang L Zhou Y Luo H Xu H. Dysregulation of ubiquitin-proteasome system in neurodegenerative diseases. Front Aging Neurosci. (2016) 8:303. doi: 10.3389/fnagi.2016.00303 28018215 PMC5156861

[44] Kim KY Stevens MV Akter MH Rusk SE Huang RJ Cohen A. Parkin is a lipid-responsive regulator of fat uptake in mice and mutant human cells. J Clin Invest. (2011) 121:3701–12. doi: 10.1172/JCI44736 PMC3171101 21865652

[45] Zechner R Zimmermann R Eichmann TO Kohlwein SD Haemmerle G Lass A. FAT SIGNALS–lipases and lipolysis in lipid metabolism and signaling. Cell Metab. (2012) 15:279–91. doi: 10.1016/j.cmet.2011.12.018 PMC3314979 22405066

[46] Yoon H Shaw JL Haigis MC Greka A. Lipid metabolism in sickness and in health: Emerging regulators of lipotoxicity. Mol Cell. (2021) 81:3708–30. doi: 10.1016/j.molcel.2021.08.027 PMC8620413 34547235

[47] Liu F Chen J Li K Li H Zhu Y Zhai Y. Ubiquitination and deubiquitination in cancer: from mechanisms to novel therapeutic approaches. Mol Cancer. (2024) 23:148. doi: 10.1186/s12943-024-02046-3 39048965 PMC11270804

[48] Yau R Rape M. The increasing complexity of the ubiquitin code. Nat Cell Biol. (2016) 18:579–86. doi: 10.1038/ncb3358 27230526

[49] Zhu S Gu H Peng C Xia F Cao H Cui H. Regulation of glucose, fatty acid and amino acid metabolism by ubiquitination and SUMOylation for cancer progression. Front Cell Dev Biol. (2022) 10:849625. doi: 10.3389/fcell.2022.849625 35392171 PMC8981989

[50] Swatek KN Usher JL Kueck AF Gladkova C Mevissen TET Pruneda JN. Insights into ubiquitin chain architecture using Ub-clipping. Nature. (2019) 572:533–7. doi: 10.1038/s41586-019-1482-y PMC6823057 31413367

[51] Behrends C Harper JW. Constructing and decoding unconventional ubiquitin chains. Nat Struct Mol Biol. (2011) 18:520–8. doi: 10.1038/nsmb.2066 21540891

[52] Wang D Zou Y Huang X Yin Z Li M Xu J. The role of SMURFs in non-cancerous diseases. FASEB J. (2023) 37:e23110. doi: 10.1096/fj.202300598R 37490283

[53] Metzger MB Hristova VA Weissman AM. HECT and RING finger families of E3 ubiquitin ligases at a glance. J Cell Sci. (2012) 125:531–7. doi: 10.1242/jcs.091777 PMC3381717 22389392

[54] Deshaies RJ Joazeiro CA. RING domain E3 ubiquitin ligases. Annu Rev Biochem. (2009) 78:399–434. doi: 10.1146/annurev.biochem.78.101807.093809 19489725

[55] Bedford L Lowe J Dick LR Mayer RJ Brownell JE. Ubiquitin-like protein conjugation and the ubiquitin-proteasome system as drug targets. Nat Rev Drug Discov. (2011) 10:29–46. doi: 10.1038/nrd3321 21151032 PMC7097807

[56] Swatek KN Komander D. Ubiquitin modifications. Cell Res. (2016) 26:399–422. doi: 10.1038/cr.2016.39 27012465 PMC4822133

[57] Gröbner SN Worst BC Weischenfeldt J Buchhalter I Kleinheinz K Rudneva VA. The landscape of genomic alterations across childhood cancers. Nature. (2018) 555:321–7. doi: 10.1038/nature25480 29489754

[58] Ma X Liu Y Liu Y Alexandrov LB Edmonson MN Gawad C. Pan-cancer genome and transcriptome analyses of 1,699 paediatric leukaemias and solid tumours. Nature. (2018) 555:371–6. doi: 10.1038/nature25795 PMC5854542 29489755

[59] Ricoult SJ Yecies JL Ben-Sahra I Manning BD. Oncogenic PI3K and K-Ras stimulate *de novo* lipid synthesis through mTORC1 and SREBP. Oncogene. (2016) 35:1250–60. doi: 10.1038/onc.2015.179 PMC4666838 26028026

[60] Swinnen JV Roskams T Joniau S Van Poppel H Oyen R Baert L. Overexpression of fatty acid synthase is an early and common event in the development of prostate cancer. Int J Cancer. (2002) 98:19–22. doi: 10.1002/ijc.10127 11857379

[61] Calvisi DF Wang C Ho C Ladu S Lee SA Mattu S. Increased lipogenesis, induced by AKT-mTORC1-RPS6 signaling, promotes development of human hepatocellular carcinoma. Gastroenterology. (2011) 140:1071–83. doi: 10.1053/j.gastro.2010.12.006 PMC3057329 21147110

[62] Oliynyk G Ruiz-Pérez MV Sainero-Alcolado L Dzieran J Zirath H Gallart-Ayala H. MYCN-enhanced oxidative and glycolytic metabolism reveals vulnerabilities for targeting neuroblastoma. iScience. (2019) 21:188–204. doi: 10.1016/j.isci.2019.10.020 31670074 PMC6889365

[63] Tech K Deshmukh M Gershon TR. Adaptations of energy metabolism during cerebellar neurogenesis are co-opted in medulloblastoma. Cancer Lett. (2015) 356:268–72. doi: 10.1016/j.canlet.2014.02.017 PMC4141892 24569090

[64] Tao L Mohammad MA Milazzo G Moreno-Smith M Patel TD Zorman B. MYCN-driven fatty acid uptake is a metabolic vulnerability in neuroblastoma. Nat Commun. (2022) 13:3728. doi: 10.1038/s41467-022-31331-2 35764645 PMC9240069

[65] Pascual G Avgustinova A Mejetta S Martín M Castellanos A Attolini CS. Targeting metastasis-initiating cells through the fatty acid receptor CD36. Nature. (2017) 541:41–5. doi: 10.1038/nature20791 27974793

[66] Jiang W Hu JW He XR Jin WL He XY. Statins: a repurposed drug to fight cancer. J Exp Clin Cancer Res. (2021) 40:241. doi: 10.1186/s13046-021-02041-2 34303383 PMC8306262

[67] Rashkovan M Albero R Gianni F Perez-Duran P Miller HI Mackey AL. Intracellular cholesterol pools regulate oncogenic signaling and epigenetic circuitries in early T-cell precursor acute lymphoblastic leukemia. Cancer Discov. (2022) 12:856–71. doi: 10.1158/2159-8290.CD-21-0551 PMC8904296 34711640

[68] Fiorentino R Chiarelli F. Statins in children, an update. Int J Mol Sci. (2023) 24. doi: 10.3390/ijms24021366 PMC9862804 36674877

[69] Wang W Chu HJ Liang YC Huang JM Shang CL Tan H. FABP5 correlates with poor prognosis and promotes tumor cell growth and metastasis in cervical cancer. Tumour Biol. (2016) 37:14873–83. doi: 10.1007/s13277-016-5350-1 27644245

[70] Sun J Esplugues E Bort A Cardelo MP Ruz-Maldonado I Fernández-Tussy P. Fatty acid binding protein 5 suppression attenuates obesity-induced hepatocellular carcinoma by promoting ferroptosis and intratumoral immune rewiring. Nat Metab. (2024) 6:741–63. doi: 10.1038/s42255-024-01019-6 PMC12355809 38664583

[71] Wang Y Wahafu A Wu W Xiang J Huo L Ma X. FABP5 enhances Malignancies of lower-grade gliomas via canonical activation of NF-κB signaling. J Cell Mol Med. (2021) 25:4487–500. doi: 10.1111/jcmm.16536 PMC8093984 33837625

[72] Fernández-García P Malet-Engra G Torres M Hanson D Rosselló CA Román R. Evolving diagnostic and treatment strategies for pediatric CNS tumors: the impact of lipid metabolism. Biomedicines. (2023) 11. doi: 10.3390/biomedicines11051365 PMC10216525 37239036

[73] Beyaz S Mana MD Roper J Kedrin D Saadatpour A Hong SJ. High-fat diet enhances stemness and tumorigenicity of intestinal progenitors. Nature. (2016) 531:53–8. doi: 10.1038/nature17173 PMC4846772 26935695

[74] Labbé DP Zadra G Yang M Reyes JM Lin CY Cacciatore S. High-fat diet fuels prostate cancer progression by rewiring the metabolome and amplifying the MYC program. Nat Commun. (2019) 10:4358. doi: 10.1038/s41467-019-12298-z 31554818 PMC6761092

[75] Asgharpour A Cazanave SC Pacana T Seneshaw M Vincent R Banini BA. A diet-induced animal model of non-alcoholic fatty liver disease and hepatocellular cancer. J Hepatol. (2016) 65:579–88. doi: 10.1016/j.jhep.2016.05.005 PMC5012902 27261415

[76] Baird J Jacob C Barker M Fall CH Hanson M Harvey NC. Developmental origins of health and disease: A lifecourse approach to the prevention of non-communicable diseases. Healthcare (Basel). (2017) 5. doi: 10.3390/healthcare5010014 PMC5371920 28282852

[77] Stacy SL Buchanich JM Ma ZQ Mair C Robertson L Sharma RK. Maternal obesity, birth size, and risk of childhood cancer development. Am J Epidemiol. (2019) 188:1503–11. doi: 10.1093/aje/kwz118 PMC6670047 31107539

[78] Specht IO Huybrechts I Frederiksen P Steliarova-Foucher E Chajes V Heitmann BL. The influence of prenatal exposure to trans-fatty acids for development of childhood haematopoietic neoplasms (EnTrance): a natural societal experiment and a case-control study. Nutr J. (2018) 17:13. doi: 10.1186/s12937-018-0317-2 29368605 PMC5784610

[79] Wood AJ Raynes-Greenow CH Carberry AE Jeffery HE. Neonatal length inaccuracies in clinical practice and related percentile discrepancies detected by a simple length-board. J Paediatr Child Health. (2013) 49:199–203. doi: 10.1111/jpc.2013.49.issue-3 23432733

[80] Bélanger V Morel S Napartuk M Bouchard I Meloche C Curnier D. Abnormal HDL lipid and protein composition following pediatric cancer treatment: an associative study. Lipids Health Dis. (2023) 22:72. doi: 10.1186/s12944-023-01822-2 37301877 PMC10257312

[81] Suh E Stratton KL Leisenring WM Nathan PC Ford JS Freyer DR. Late mortality and chronic health conditions in long-term survivors of early-adolescent and young adult cancers: a retrospective cohort analysis from the Childhood Cancer Survivor Study. Lancet Oncol. (2020) 21:421–35. doi: 10.1016/S1470-2045(19)30800-9 PMC7392388 32066543

[82] Moke DJ Hamilton AS Chehab L Deapen D Freyer DR. Obesity and risk for second Malignant neoplasms in childhood cancer survivors: A case-control study utilizing the california cancer registry. Cancer Epidemiol Biomarkers Prev. (2019) 28:1612–20. doi: 10.1158/1055-9965.EPI-19-0466 PMC6774883 31337641

[83] Priem B van Leent MMT Teunissen AJP Sofias AM Mourits VP Willemsen L. Trained immunity-promoting nanobiologic therapy suppresses tumor growth and potentiates checkpoint inhibition. Cell. (2020) 183:786–801.e19. doi: 10.1016/j.cell.2020.09.059 33125893 PMC8074872

[84] Liu X Bao X Hu M Chang H Jiao M Cheng J. Inhibition of PCSK9 potentiates immune checkpoint therapy for cancer. Nature. (2020) 588:693–8. doi: 10.1038/s41586-020-2911-7 PMC7770056 33177715

[85] Kopecka J Godel M Riganti C. Cholesterol metabolism: At the cross road between cancer cells and immune environment. Int J Biochem Cell Biol. (2020) 129:105876. doi: 10.1016/j.biocel.2020.105876 33166677

[86] Yan A Jia Z Qiao C Wang M Ding X. Cholesterol metabolism in drug−resistant cancer (Review). Int J Oncol. (2020) 57:1103–15. doi: 10.3892/ijo.2020.5124 33491740

[87] Dessì S Batetta B Spano O Sanna F Tonello M Giacchino M. Clinical remission is associated with restoration of normal high-density lipoprotein cholesterol levels in children with Malignancies. Clin Sci (Lond). (1995) 89:505–10. doi: 10.1042/cs0890505 8549065

[88] Bosteels V Maréchal S De Nolf C Rennen S Maelfait J Tavernier SJ. LXR signaling controls homeostatic dendritic cell maturation. Sci Immunol. (2023) 8:eadd3955. doi: 10.1126/sciimmunol.add3955 37172103

[89] Min J Wu Y Huang S Li Y Lv X Tang R. Serum cholesterol level as a predictive biomarker for prognosis of Neuroblastoma. BMC Pediatr. (2024) 24:205. doi: 10.1186/s12887-024-04700-7 38519890 PMC10958969

[90] Tran GB Ding J Ye B Liu M Yu Y Zha Y. Caffeine supplementation and FOXM1 inhibition enhance the antitumor effect of statins in neuroblastoma. Cancer Res. (2023) 83:2248–61. doi: 10.1158/0008-5472.CAN-22-3450 PMC10320471 37057874

[91] He Y Cui X Lin Y Wang Y Wu D Fang Y. Using elevated cholesterol synthesis as a prognostic marker in wilms' Tumor: A bioinformatic analysis. BioMed Res Int. (2021) 2021:8826286. doi: 10.1155/2021/8826286 33628817 PMC7886595

[92] Gordon RE Zhang L Peri S Kuo YM Du F Egleston BL. Statins synergize with hedgehog pathway inhibitors for treatment of medulloblastoma. Clin Cancer Res. (2018) 24:1375–88. doi: 10.1158/1078-0432.CCR-17-2923 PMC5856627 29437795

[93] Sharpe LJ Cook EC Zelcer N Brown AJ. The UPS and downs of cholesterol homeostasis. Trends Biochem Sci. (2014) 39:527–35. doi: 10.1016/j.tibs.2014.08.008 25220377

[94] Liu M Xia Y Ding J Ye B Zhao E Choi JH. Transcriptional profiling reveals a common metabolic program in high-risk human neuroblastoma and mouse neuroblastoma sphere-forming cells. Cell Rep. (2016) 17:609–23. doi: 10.1016/j.celrep.2016.09.021 PMC5536348 27705805

[95] van den Boomen DJH Volkmar N Lehner PJ. Ubiquitin-mediated regulation of sterol homeostasis. Curr Opin Cell Biol. (2020) 65:103–11. doi: 10.1016/j.ceb.2020.04.010 32580085

[96] Cheng C Ru P Geng F Liu J Yoo JY Wu X. Glucose-mediated N-glycosylation of SCAP is essential for SREBP-1 activation and tumor growth. Cancer Cell. (2015) 28:569–81. doi: 10.1016/j.ccell.2015.09.021 PMC4643405 26555173

[97] Gu D Zhou F You H Gao J Kang T Dixit D. Sterol regulatory element-binding protein 2 maintains glioblastoma stem cells by keeping the balance between cholesterol biosynthesis and uptake. Neuro Oncol. (2023) 25:1578–91. doi: 10.1093/neuonc/noad060 PMC10651206 36934350

[98] Lee SY Slagle-Webb B Schengrund CL Zhu J Connor JR. Association between iron and cholesterol in neuroblastomas. Anticancer Res. (2021) 41:2795–804. doi: 10.21873/anticanres.15060 34083269

[99] Drabkin HA Gemmill RM. Cholesterol and the development of clear-cell renal carcinoma. Curr Opin Pharmacol. (2012) 12:742–50. doi: 10.1016/j.coph.2012.08.002 22939900

[100] Gemmill RM West JD Boldog F Tanaka N Robinson LJ Smith DI. The hereditary renal cell carcinoma 3;8 translocation fuses FHIT to a patched-related gene, TRC8. Proc Natl Acad Sci U S A. (1998) 95:9572–7. doi: 10.1073/pnas.95.16.9572 PMC21380 9689122

[101] Irisawa M Inoue J Ozawa N Mori K Sato R. The sterol-sensing endoplasmic reticulum (ER) membrane protein TRC8 hampers ER to Golgi transport of sterol regulatory element-binding protein-2 (SREBP-2)/SREBP cleavage-activated protein and reduces SREBP-2 cleavage. J Biol Chem. (2009) 284:28995–9004. doi: 10.1074/jbc.M109.041376 PMC2781446 19706601

[102] Zhang L Rajbhandari P Priest C Sandhu J Wu X Temel R. Inhibition of cholesterol biosynthesis through RNF145-dependent ubiquitination of SCAP. Elife. (2017) 6. doi: 10.7554/eLife.28766 PMC5656429 29068315

[103] Song BL Sever N DeBose-Boyd RA. Gp78, a membrane-anchored ubiquitin ligase, associates with Insig-1 and couples sterol-regulated ubiquitination to degradation of HMG CoA reductase. Mol Cell. (2005) 19:829–40. doi: 10.1016/j.molcel.2005.08.009 16168377

[104] Lee JN Gong Y Zhang X Ye J. Proteasomal degradation of ubiquitinated Insig proteins is determined by serine residues flanking ubiquitinated lysines. Proc Natl Acad Sci U S A. (2006) 103:4958–63. doi: 10.1073/pnas.0600422103 PMC1405624 16549805

[105] Zhou ZS Li MX Liu J Jiao H Xia JM Shi XJ. Competitive oxidation and ubiquitylation on the evolutionarily conserved cysteine confer tissue-specific stabilization of Insig-2. Nat Commun. (2020) 11:379. doi: 10.1038/s41467-019-14231-w 31953408 PMC6969111

[106] Maier CR Hartmann O Prieto-Garcia C Al-Shami KM Schlicker L Vogel FCE. USP28 controls SREBP2 and the mevalonate pathway to drive tumour growth in squamous cancer. Cell Death Differ. (2023) 30:1710–25. doi: 10.1038/s41418-023-01173-6 PMC10307777 37202505

[107] Sorrentino V Zelcer N. Post-transcriptional regulation of lipoprotein receptors by the E3-ubiquitin ligase inducible degrader of the low-density lipoprotein receptor. Curr Opin Lipidol. (2012) 23:213–9. doi: 10.1097/MOL.0b013e3283532947 22510808

[108] Zelcer N Hong C Boyadjian R Tontonoz P. LXR regulates cholesterol uptake through Idol-dependent ubiquitination of the LDL receptor. Science. (2009) 325:100–4. doi: 10.1126/science.1168974 PMC2777523 19520913

[109] Cook EC Nelson JK Sorrentino V Koenis D Moeton M Scheij S. Identification of the ER-resident E3 ubiquitin ligase RNF145 as a novel LXR-regulated gene. PloS One. (2017) 12:e0172721. doi: 10.1371/journal.pone.0172721 28231341 PMC5322959

[110] Okabe A Urano Y Itoh S Suda N Kotani R Nishimura Y. Adaptive responses induced by 24S-hydroxycholesterol through liver X receptor pathway reduce 7-ketocholesterol-caused neuronal cell death. Redox Biol. (2013) 2:28–35. doi: 10.1016/j.redox.2013.11.007 24371802 PMC3871289

[111] Yuan B Shi K Zha J Cai Y Gu Y Huang K. Nuclear receptor modulators inhibit osteosarcoma cell proliferation and tumour growth by regulating the mTOR signaling pathway. Cell Death Dis. (2023) 14:51. doi: 10.1038/s41419-022-05545-7 36681687 PMC9867777

[112] Li X Zhang S Blander G Tse JG Krieger M Guarente L. SIRT1 deacetylates and positively regulates the nuclear receptor LXR. Mol Cell. (2007) 28:91–106. doi: 10.1016/j.molcel.2007.07.032 17936707

[113] Dong Y Lathrop W Weaver D Qiu Q Cini J Bertolini D. Molecular cloning and characterization of LR3, a novel LDL receptor family protein with mitogenic activity. Biochem Biophys Res Commun. (1998) 251:784–90. doi: 10.1006/bbrc.1998.9545 9790987

[114] Ma KL Liu J Wang CX Ni J Zhang Y Wu Y. Activation of mTOR modulates SREBP-2 to induce foam cell formation through increased retinoblastoma protein phosphorylation. Cardiovasc Res. (2013) 100:450–60. doi: 10.1093/cvr/cvt203 24068000

[115] Zhao X Feng D Wang Q Abdulla A Xie XJ Zhou J. Regulation of lipogenesis by cyclin-dependent kinase 8-mediated control of SREBP-1. J Clin Invest. (2012) 122:2417–27. doi: 10.1172/JCI61462 PMC3386818 22684109

[116] Gill S Stevenson J Kristiana I Brown AJ. Cholesterol-dependent degradation of squalene monooxygenase, a control point in cholesterol synthesis beyond HMG-CoA reductase. Cell Metab. (2011) 13:260–73. doi: 10.1016/j.cmet.2011.01.015 21356516

[117] Kikkert M Doolman R Dai M Avner R Hassink G van Voorden S. Human HRD1 is an E3 ubiquitin ligase involved in degradation of proteins from the endoplasmic reticulum. J Biol Chem. (2004) 279:3525–34. doi: 10.1074/jbc.M307453200 14593114

[118] Raal FJ Giugliano RP Sabatine MS Koren MJ Blom D Seidah NG. PCSK9 inhibition-mediated reduction in Lp(a) with evolocumab: an analysis of 10 clinical trials and the LDL receptor's role. J Lipid Res. (2016) 57:1086–96. doi: 10.1194/jlr.P065334 PMC4878192 27102113

[119] Romagnuolo R Scipione CA Boffa MB Marcovina SM Seidah NG Koschinsky ML. Lipoprotein(a) catabolism is regulated by proprotein convertase subtilisin/kexin type 9 through the low density lipoprotein receptor. J Biol Chem. (2015) 290:11649–62. doi: 10.1074/jbc.M114.611988 PMC4416867 25778403

[120] Blaeschke F Paul MC Schuhmann MU Rabsteyn A Schroeder C Casadei N. Low mutational load in pediatric medulloblastoma still translates into neoantigens as targets for specific T-cell immunotherapy. Cytotherapy. (2019) 21:973–86. doi: 10.1016/j.jcyt.2019.06.009 31351799

[121] Yuan J Cai T Zheng X Ren Y Qi J Lu X. Potentiating CD8(+) T cell antitumor activity by inhibiting PCSK9 to promote LDLR-mediated TCR recycling and signaling. Protein Cell. (2021) 12:240–60. doi: 10.1007/s13238-021-00821-2 PMC8018994 33606190

[122] Loregger A Raaben M Tan J Scheij S Moeton M van den Berg M. Haploid mammalian genetic screen identifies UBXD8 as a key determinant of HMGCR degradation and cholesterol biosynthesis. Arterioscler Thromb Vasc Biol. (2017) 37:2064–74. doi: 10.1161/ATVBAHA.117.310002 PMC5671778 28882874

[123] Menzies SA Volkmar N van den Boomen DJ Timms RT Dickson AS Nathan JA. The sterol-responsive RNF145 E3 ubiquitin ligase mediates the degradation of HMG-CoA reductase together with gp78 and Hrd1. Elife. (2018) 7. doi: 10.7554/eLife.40009 PMC6292692 30543180

[124] Sever N Yang T Brown MS Goldstein JL DeBose-Boyd RA. Accelerated degradation of HMG CoA reductase mediated by binding of insig-1 to its sterol-sensing domain. Mol Cell. (2003) 11:25–33. doi: 10.1016/S1097-2765(02)00822-5 12535518

[125] Sever N Song BL Yabe D Goldstein JL Brown MS DeBose-Boyd RA. Insig-dependent ubiquitination and degradation of mammalian 3-hydroxy-3-methylglutaryl-CoA reductase stimulated by sterols and geranylgeraniol. J Biol Chem. (2003) 278:52479–90. doi: 10.1074/jbc.M310053200 14563840

[126] Lu XY Shi XJ Hu A Wang JQ Ding Y Jiang W. Feeding induces cholesterol biosynthesis via the mTORC1-USP20-HMGCR axis. Nature. (2020) 588:479–84. doi: 10.1038/s41586-020-2928-y 33177714

[127] Marcuzzi A Zanin V Piscianz E Tricarico PM Vuch J Girardelli M. Lovastatin-induced apoptosis is modulated by geranylgeraniol in a neuroblastoma cell line. Int J Dev Neurosci. (2012) 30:451–6. doi: 10.1016/j.ijdevneu.2012.06.002 22759742

[128] Stokes ME Small JC Vasciaveo A Shimada K Hirschhorn T Califano A. Mesenchymal subtype neuroblastomas are addicted to TGF-βR2/HMGCR-driven protein geranylgeranylation. Sci Rep. (2020) 10:10748. doi: 10.1038/s41598-020-67310-0 32612149 PMC7329873

[129] Moriceau G Roelofs AJ Brion R Redini F Ebetion FH Rogers MJ. Synergistic inhibitory effect of apomine and lovastatin on osteosarcoma cell growth. Cancer. (2012) 118:750–60. doi: 10.1002/cncr.v118.3 21751201

[130] Codenotti S Zizioli D Mignani L Rezzola S Tabellini G Parolini S. Hyperactive akt1 signaling increases tumor progression and DNA repair in embryonal rhabdomyosarcoma RD line and confers susceptibility to glycolysis and mevalonate pathway inhibitors. Cells. (2022) 11. doi: 10.3390/cells11182859 PMC9497225 36139434

[131] Du X Ou Y Zhang M Li K Huang W Jiang D. The mevalonate pathway promotes the metastasis of osteosarcoma by regulating YAP1 activity via RhoA. Genes Dis. (2022) 9:741–52. doi: 10.1016/j.gendis.2020.11.009 PMC9243346 35782968

[132] Jakobisiak M Golab J. Potential antitumor effects of statins (Review). Int J Oncol. (2003) 23:1055–69. doi: 10.3892/ijo.23.4.1055 12963986

[133] Wang Y Ma X Xu E Huang Z Yang C Zhu K. Identifying squalene epoxidase as a metabolic vulnerability in high-risk osteosarcoma using an artificial intelligence-derived prognostic index. Clin Transl Med. (2024) 14:e1586. doi: 10.1002/ctm2.v14.2 38372422 PMC10875711

[134] Jiang YC Xu QT Wang HB Ren SY Zhang Y. A novel prognostic signature related to programmed cell death in osteosarcoma. Front Immunol. (2024) 15:1427661. doi: 10.3389/fimmu.2024.1427661 39015570 PMC11250594

[135] Ji Y Liu L Liu Y Ma Y Ji Z Wu X. Exploring gene biomarkers and targeted drugs for ferroptosis and cuproptosis in osteosarcoma: A bioinformatic approach. Environ Toxicol. (2024). doi: 10.1002/tox.24250 PMC12069744 38546286

[136] Jiang X Stockwell BR Conrad M. Ferroptosis: mechanisms, biology and role in disease. Nat Rev Mol Cell Biol. (2021) 22:266–82. doi: 10.1038/s41580-020-00324-8 PMC8142022 33495651

[137] Tsvetkov P Coy S Petrova B Dreishpoon M Verma A Abdusamad M. Copper induces cell death by targeting lipoylated TCA cycle proteins. Science. (2022) 375:1254–61. doi: 10.1126/science.abf0529 PMC9273333 35298263

[138] Cen D Brayton D Shahandeh B Meyskens FL Farmer PJ. Disulfiram facilitates intracellular Cu uptake and induces apoptosis in human melanoma cells. J Med Chem. (2004) 47:6914–20. doi: 10.1021/jm049568z 15615540

[139] Chen D Cui QC Yang H Dou QP. Disulfiram, a clinically used anti-alcoholism drug and copper-binding agent, induces apoptotic cell death in breast cancer cultures and xenografts via inhibition of the proteasome activity. Cancer Res. (2006) 66:10425–33. doi: 10.1158/0008-5472.CAN-06-2126 17079463

[140] O'Day S Phase II. randomized, controlled, double-blinded trial of weekly elesclomol plus paclitaxel versus paclitaxel alone for stage IV metastatic melanoma. J Clin Oncol. (2009) 27:5452–8. doi: 10.1200/JCO.2008.17.1579 19826135

[141] Scott NA Sharpe LJ Capell-Hattam IM Gullo SJ Luu W Brown AJ. The cholesterol synthesis enzyme lanosterol 14α-demethylase is post-translationally regulated by the E3 ubiquitin ligase MARCH6. Biochem J. (2020) 477:541–55. doi: 10.1042/BCJ20190647 PMC6993871 31904814

[142] Qian L Scott NA Capell-Hattam IM Draper EA Fenton NM Luu W. Cholesterol synthesis enzyme SC4MOL is fine-tuned by sterols and targeted for degradation by the E3 ligase MARCHF6. J Lipid Res. (2023) 64:100362. doi: 10.1016/j.jlr.2023.100362 36958722 PMC10176258

[143] Stevenson J Luu W Kristiana I Brown AJ. Squalene mono-oxygenase, a key enzyme in cholesterol synthesis, is stabilized by unsaturated fatty acids. Biochem J. (2014) 461:435–42. doi: 10.1042/BJ20131404 24840124

[144] Calkin AC Tontonoz P. Transcriptional integration of metabolism by the nuclear sterol-activated receptors LXR and FXR. Nat Rev Mol Cell Biol. (2012) 13:213–24. doi: 10.1038/nrm3312 PMC3597092 22414897

[145] Belisario DC Akman M Godel M Campani V Patrizio MP Scotti L. ABCA1/ABCB1 ratio determines chemo- and immune-sensitivity in human osteosarcoma. Cells. (2020) 9. doi: 10.3390/cells9030647 PMC7140509 32155954

[146] Ingram WJ Crowther LM Little EB Freeman R Harliwong I Veleva D. ABC transporter activity linked to radiation resistance and molecular subtype in pediatric medulloblastoma. Exp Hematol Oncol. (2013) 2:26. doi: 10.1186/2162-3619-2-26 24219920 PMC3851566

[147] Roundhill EA Jabri S Burchill SA. ABCG1 and Pgp identify drug resistant, self-renewing osteosarcoma cells. Cancer Lett. (2019) 453:142–57. doi: 10.1016/j.canlet.2019.03.011 30910588

[148] Ogura M Ayaori M Terao Y Hisada T Iizuka M Takiguchi S. Proteasomal inhibition promotes ATP-binding cassette transporter A1 (ABCA1) and ABCG1 expression and cholesterol efflux from macrophages *in vitro* and *in vivo* . Arterioscler Thromb Vasc Biol. (2011) 31:1980–7. doi: 10.1161/ATVBAHA.111.228478 21817095

[149] Hsieh V Kim MJ Gelissen IC Brown AJ Sandoval C Hallab JC. Cellular cholesterol regulates ubiquitination and degradation of the cholesterol export proteins ABCA1 and ABCG1. J Biol Chem. (2014) 289:7524–36. doi: 10.1074/jbc.M113.515890 PMC3953266 24500716

[150] Raghavan S Singh NK Mani AM Rao GN. Protease-activated receptor 1 inhibits cholesterol efflux and promotes atherogenesis via cullin 3-mediated degradation of the ABCA1 transporter. J Biol Chem. (2018) 293:10574–89. doi: 10.1074/jbc.RA118.003491 PMC6036195 29777060

[151] Boro M Govatati S Kumar R Singh NK Pichavaram P Traylor JG. Thrombin-Par1 signaling axis disrupts COP9 signalosome subunit 3-mediated ABCA1 stabilization in inducing foam cell formation and atherogenesis. Cell Death Differ. (2021) 28:780–98. doi: 10.1038/s41418-020-00623-9 PMC7862243 32968199

[152] Wang K Liu X. Determining the effects of neddylation on cullin-RING ligase-dependent protein ubiquitination. Curr Protoc. (2022) 2:e401. doi: 10.1002/cpz1.v2.3 35316580 PMC8969890

[153] Rao F Lin H Su Y. Cullin-RING ligase regulation by the COP9 signalosome: structural mechanisms and new physiologic players. Adv Exp Med Biol. (2020) 1217:47–60. doi: 10.1002/cpz1.401 31898221

[154] Suzuki S Shuto T Sato T Kaneko M Takada T Suico MA. Inhibition of post-translational N-glycosylation by HRD1 that controls the fate of ABCG5/8 transporter. Sci Rep. (2014) 4:4258. doi: 10.1038/srep04258 24584735 PMC3939451

[155] Tan J Liang H Yang B Zhu S Wu G Li L. Identification and analysis of three hub prognostic genes related to osteosarcoma metastasis. J Oncol. (2021) 2021:6646459. doi: 10.1155/2021/6646459 33564309 PMC7867449

[156] Pasello M Giudice AM Cristalli C Manara MC Mancarella C Parra A. ABCA6 affects the Malignancy of Ewing sarcoma cells via cholesterol-guided inhibition of the IGF1R/AKT/MDM2 axis. Cell Oncol (Dordr). (2022) 45:1237–51. doi: 10.1007/s13402-022-00713-5 PMC9747862 36149602

[157] Lamers F Schild L Koster J Versteeg R Caron HN Molenaar JJ. Targeted BIRC5 silencing using YM155 causes cell death in neuroblastoma cells with low ABCB1 expression. Eur J Cancer. (2012) 48:763–71. doi: 10.1016/j.ejca.2011.10.012 22088485

[158] Chen Y Zhu L Ji L Yang Y Lu L Wang X. Silencing the ACAT1 gene in human SH-SY5Y neuroblastoma cells inhibits the expression of cyclo-oxygenase 2 (COX2) and reduces β-amyloid-induced toxicity due to activation of protein kinase C (PKC) and ERK. Med Sci Monit. (2018) 24:9007–18. doi: 10.12659/MSM.912862 PMC6299791 30541014

[159] Jeng I Klemm N. Acyl-CoA cholesterol acyltransferase in cultured glioblastoma cells. Neurochem Res. (1984) 9:1193–210. doi: 10.1007/BF00973034 6504235

[160] Yang W Bai Y Xiong Y Zhang J Chen S Zheng X. Potentiating the antitumour response of CD8(+) T cells by modulating cholesterol metabolism. Nature. (2016) 531:651–5. doi: 10.1038/nature17412 PMC4851431 26982734

[161] Wang YJ Bian Y Luo J Lu M Xiong Y Guo SY. Cholesterol and fatty acids regulate cysteine ubiquitylation of ACAT2 through competitive oxidation. Nat Cell Biol. (2017) 19:808–19. doi: 10.1038/ncb3551 PMC5518634 28604676

[162] Zhu Y Gu L Lin X Zhou X Lu B Liu C. P53 deficiency affects cholesterol esterification to exacerbate hepatocarcinogenesis. Hepatology. (2023) 77:1499–511. doi: 10.1002/hep.32518 PMC11186660 35398929

[163] Danielli M Perne L Jarc Jovičić E Petan T. Lipid droplets and polyunsaturated fatty acid trafficking: Balancing life and death. Front Cell Dev Biol. (2023) 11:1104725. doi: 10.3389/fcell.2023.1104725 36776554 PMC9911892

[164] Zhang C Liu J Huang G Zhao Y Yue X Wu H. Cullin3-KLHL25 ubiquitin ligase targets ACLY for degradation to inhibit lipid synthesis and tumor progression. Genes Dev. (2016) 30:1956–70. doi: 10.1101/gad.283283.116 PMC5066239 27664236

[165] Han C Yang L Choi HH Baddour J Achreja A Liu Y. Amplification of USP13 drives ovarian cancer metabolism. Nat Commun. (2016) 7:13525. doi: 10.1038/ncomms13525 27892457 PMC5133706

[166] Gu L Zhu Y Lin X Lu B Zhou F Zhou F. The IKKβ-USP30-ACLY axis controls lipogenesis and tumorigenesis. Hepatology. (2021) 73:160–74. doi: 10.1002/hep.31249 32221968

[167] Zhu G Xia Y Zhao Z Li A Li H Xiao T. LncRNA XIST from the bone marrow mesenchymal stem cell derived exosome promotes osteosarcoma growth and metastasis through miR-655/ACLY signal. Cancer Cell Int. (2022) 22:330. doi: 10.1186/s12935-022-02746-0 36309693 PMC9617450

[168] Xin M Qiao Z Li J Liu J Song S Zhao X. miR-22 inhibits tumor growth and metastasis by targeting ATP citrate lyase: evidence in osteosarcoma, prostate cancer, cervical cancer and lung cancer. Oncotarget. (2016) 7:44252–65. doi: 10.18632/oncotarget.10020 PMC5190093 27317765

[169] Qi L Heredia JE Altarejos JY Screaton R Goebel N Niessen S. TRB3 links the E3 ubiquitin ligase COP1 to lipid metabolism. Science. (2006) 312:1763–6. doi: 10.1126/science.1123374 16794074

[170] Ma J Yan R Zu X Cheng JM Rao K Liao DF. Aldo-keto reductase family 1 B10 affects fatty acid synthesis by regulating the stability of acetyl-CoA carboxylase-alpha in breast cancer cells. J Biol Chem. (2008) 283:3418–23. doi: 10.1074/jbc.M707650200 18056116

[171] Matsunaga T Wada Y Endo S Soda M El-Kabbani O Hara A. Aldo-keto reductase 1B10 and its role in proliferation capacity of drug-resistant cancers. Front Pharmacol. (2012) 3:5. doi: 10.3389/fphar.2012.00005 22319498 PMC3269042

[172] Asturias FJ Chadick JZ Cheung IK Stark H Witkowski A Joshi AK. Structure and molecular organization of mammalian fatty acid synthase. Nat Struct Mol Biol. (2005) 12:225–32. doi: 10.1038/nsmb899 15711565

[173] Sun T Zhong X Song H Liu J Li J Leung F. Anoikis resistant mediated by FASN promoted growth and metastasis of osteosarcoma. Cell Death Dis. (2019) 10:298. doi: 10.1038/s41419-019-1532-2 30931932 PMC6443797

[174] Sheeter DA Garza S Park HG Benhamou LE Badi NR Espinosa EC. Unsaturated fatty acid synthesis is associated with worse survival and is differentially regulated by MYCN and tumor suppressor microRNAs in neuroblastoma. Cancers (Basel). (2024) 16. doi: 10.3390/cancers16081590 PMC11048984 38672672

[175] Qin XY Su T Yu W Kojima S. Lipid desaturation-associated endoplasmic reticulum stress regulates MYCN gene expression in hepatocellular carcinoma cells. Cell Death Dis. (2020) 11:66. doi: 10.1038/s41419-020-2257-y 31988297 PMC6985230

[176] Chen XY Ruan HB Long XH Peng AF Zhou LD Liu JM. Blocking fatty acid synthase inhibits tumor progression of human osteosarcoma by regulating the human epidermal growth factor receptor 2/phosphoinositide 3-kinase/protein kinase B signaling pathway in xenograft models. Exp Ther Med. (2017) 13:2411–6. doi: 10.3892/etm.2017.4284 PMC5443167 28565856

[177] Bhatia B Hsieh M Kenney AM Nahlé Z. Mitogenic Sonic hedgehog signaling drives E2F1-dependent lipogenesis in progenitor cells and medulloblastoma. Oncogene. (2011) 30:410–22. doi: 10.1038/onc.2010.454 PMC3072890 20890301

[178] Graner E Tang D Rossi S Baron A Migita T Weinstein LJ. The isopeptidase USP2a regulates the stability of fatty acid synthase in prostate cancer. Cancer Cell. (2004) 5:253–61. doi: 10.1016/S1535-6108(04)00055-8 15050917

[179] Liu H Gan Q Lai Y Pan Z Jin Q Li J. USP14 increases the sensitivity of retinoblastoma to cisplatin by mediating the ferroptosis. Naunyn Schmiedebergs Arch Pharmacol. (2024) 397:8671–80. doi: 10.1007/s00210-024-03174-9 PMC11522062 38819674

[180] Wang CL Wang JY Liu ZY Ma XM Wang XW Jin H. Ubiquitin-specific protease 2a stabilizes MDM4 and facilitates the p53-mediated intrinsic apoptotic pathway in glioblastoma. Carcinogenesis. (2014) 35:1500–9. doi: 10.1093/carcin/bgu015 24445145

[181] Smith J Su X El-Maghrabi R Stahl PD Abumrad NA. Opposite regulation of CD36 ubiquitination by fatty acids and insulin: effects on fatty acid uptake. J Biol Chem. (2008) 283:13578–85. doi: 10.1074/jbc.M800008200 PMC2376227 18353783

[182] Xia X Xu Q Liu M Chen X Liu X He J. Deubiquitination of CD36 by UCHL1 promotes foam cell formation. Cell Death Dis. (2020) 11:636. doi: 10.1038/s41419-020-02888-x 32801299 PMC7429868

[183] Wang H Franco F Tsui YC Xie X Trefny MP Zappasodi R. CD36-mediated metabolic adaptation supports regulatory T cell survival and function in tumors. Nat Immunol. (2020) 21:298–308. doi: 10.1038/s41590-019-0589-5 32066953 PMC7043937

[184] Jian YK Zhu HY Wu XL Li B. Thrombospondin 1 triggers osteosarcoma cell metastasis and tumor angiogenesis. Oncol Res. (2019) 27:211–8. doi: 10.3727/096504018X15208993118389 PMC7848452 29540257

[185] Yamazaki H Handa A Nishi M Tokunaga T Tomisawa M Hatanaka H. Ribozyme mediated down-regulation of thrombospondin receptor CD36 inhibits the growth of the human osteosarcoma cell line. Oncol Rep. (2004) 11:371–4. doi: 10.3892/or.11.2.371 14719070

[186] Zhang N Han Y Cao H Wang Q. Inflammasome-related gene signatures as prognostic biomarkers in osteosarcoma. J Cell Mol Med. (2024) 28:e18286. doi: 10.1111/jcmm.18286 38742843 PMC11092527

[187] Yang M Su Y Xu K Zheng H Cai Y Wen P. Develop a novel signature to predict the survival and affect the immune microenvironment of osteosarcoma patients: anoikis-related genes. J Immunol Res. (2024) 2024:6595252. doi: 10.1155/2024/6595252 39431237 PMC11491172

[188] Urso CJ Zhou H. Role of CD36 in palmitic acid lipotoxicity in neuro-2a neuroblastoma cells. Biomolecules. (2021) 11. doi: 10.3390/biom11111567 PMC8615720 34827565

[189] Hale JS Otvos B Sinyuk M Alvarado AG Hitomi M Stoltz K. Cancer stem cell-specific scavenger receptor CD36 drives glioblastoma progression. Stem Cells. (2014) 32:1746–58. doi: 10.1002/stem.1716 PMC4063873 24737733

[190] Jiang M Wu N Xu B Chu Y Li X Su S. Fatty acid-induced CD36 expression via O-GlcNAcylation drives gastric cancer metastasis. Theranostics. (2019) 9:5359–73. doi: 10.7150/thno.34024 PMC6691574 31410220

[191] Miao L Zhuo Z Tang J Huang X Liu J Wang HY. FABP4 deactivates NF-κB-IL1α pathway by ubiquitinating ATPB in tumor-associated macrophages and promotes neuroblastoma progression. Clin Transl Med. (2021) 11:e395. doi: 10.1002/ctm2.v11.4 33931964 PMC8087928

[192] Zaatiti H Abdallah J Nasr Z Khazen G Sandler A Abou-Antoun TJ. Tumorigenic proteins upregulated in the MYCN-amplified IMR-32 human neuroblastoma cells promote proliferation and migration. Int J Oncol. (2018) 52:787–803. doi: 10.3892/ijo.2018.4236 29328367 PMC5807036

[193] Yang M Wei R Zhang S Hu S Liang X Yang Z. NSUN2 promotes osteosarcoma progression by enhancing the stability of FABP5 mRNA via m(5)C methylation. Cell Death Dis. (2023) 14:125. doi: 10.1038/s41419-023-05646-x 36792587 PMC9932088

[194] Zhou Y Yang D Yang Q Lv X Huang W Zhou Z. Single-cell RNA landscape of intratumoral heterogeneity and immunosuppressive microenvironment in advanced osteosarcoma. Nat Commun. (2020) 11:6322. doi: 10.1038/s41467-020-20059-6 33303760 PMC7730477

[195] Cai F Liu L Bo Y Yan W Tao X Peng Y. LncRNA RPARP-AS1 promotes the progression of osteosarcoma cells through regulating lipid metabolism. BMC Cancer. (2024) 24:166. doi: 10.1186/s12885-024-11901-x 38308235 PMC10835925

[196] Zhang S Liu H Li H Wu M Yu Y Li F. Differential CRABP-II and FABP5 expression patterns and implications for medulloblastoma retinoic acid sensitivity. RSC Adv. (2018) 8:14048–55. doi: 10.1039/C8RA00744F PMC9079906 35539303

[197] Nimmakayala RK Leon F Rachagani S Rauth S Nallasamy P Marimuthu S. Metabolic programming of distinct cancer stem cells promotes metastasis of pancreatic ductal adenocarcinoma. Oncogene. (2021) 40:215–31. doi: 10.1038/s41388-020-01518-2 PMC10041665 33110235

[198] Qu H Jiang J Zhan X Liang Y Guo Q Liu P. Integrating artificial intelligence in osteosarcoma prognosis: the prognostic significance of SERPINE2 and CPT1B biomarkers. Sci Rep. (2024) 14:4318. doi: 10.1038/s41598-024-54222-6 38383657 PMC10881519

[199] Guo X Wang A Wang W Wang Y Chen H Liu X. HRD1 inhibits fatty acid oxidation and tumorigenesis by ubiquitinating CPT2 in triple-negative breast cancer. Mol Oncol. (2021) 15:642–56. doi: 10.1002/1878-0261.12856 PMC7858277 33207079

[200] Wang W Guan J Zong Y Zhang Q Zhang W Wang H. Proteomics analysis of lipid droplets in mouse neuroblastoma cells. Acta Biochim Pol. (2022) 69:399–407. doi: 10.18388/abp.2020_5851 35616622

[201] Lam T Harmancey R Vasquez H Gilbert B Patel N Hariharan V. Reversal of intramyocellular lipid accumulation by lipophagy and a p62-mediated pathway. Cell Death Discov. (2016) 2:16061. doi: 10.1038/cddiscovery.2016.61 27625792 PMC4993124

[202] Hooper C Puttamadappa SS Loring Z Shekhtman A Bakowska JC. Spartin activates atrophin-1-interacting protein 4 (AIP4) E3 ubiquitin ligase and promotes ubiquitination of adipophilin on lipid droplets. BMC Biol. (2010) 8:72. doi: 10.1186/1741-7007-8-72 20504295 PMC2887783

[203] Filali-Mouncef Y Hunter C Roccio F Zagkou S Dupont N Primard C. The ménage à trois of autophagy, lipid droplets and liver disease. Autophagy. (2022) 18:50–72. doi: 10.1080/15548627.2021.1895658 33794741 PMC8865253

[204] Nian Z Sun Z Yu L Toh SY Sang J Li P. Fat-specific protein 27 undergoes ubiquitin-dependent degradation regulated by triacylglycerol synthesis and lipid droplet formation. J Biol Chem. (2010) 285:9604–15. doi: 10.1074/jbc.M109.043786 PMC2843210 20089860

[205] Ghosh M Niyogi S Bhattacharyya M Adak M Nayak DK Chakrabarti S. Ubiquitin ligase COP1 controls hepatic fat metabolism by targeting ATGL for degradation. Diabetes. (2016) 65:3561–72. doi: 10.2337/db16-0506 27658392

[206] Sugihara M Morito D Ainuki S Hirano Y Ogino K Kitamura A. The AAA+ ATPase/ubiquitin ligase mysterin stabilizes cytoplasmic lipid droplets. J Cell Biol. (2019) 218:949–60. doi: 10.1083/jcb.201712120 PMC6400562 30705059

[207] Wang X Ye M Wu M Fang H Xiao B Xie L. RNF213 suppresses carcinogenesis in glioblastoma by affecting MAPK/JNK signaling pathway. Clin Transl Oncol. (2020) 22:1506–16. doi: 10.1007/s12094-020-02286-x 31953610

[208] Olzmann JA Richter CM Kopito RR. Spatial regulation of UBXD8 and p97/VCP controls ATGL-mediated lipid droplet turnover. Proc Natl Acad Sci U S A. (2013) 110:1345–50. doi: 10.1073/pnas.1213738110 PMC3557085 23297223

[209] Atashi HA Arani HZ Shekarriz A Nazari H Zabolian A Rakhshan R. Cyanidin 3-O-glucoside induces the apoptosis in the osteosarcoma cells through upregulation of the PPARγ and P21: an *in vitro* study. Anticancer Agents Med Chem. (2020) 20:1087–93. doi: 10.2174/1871520620666200408081111 32268872

[210] Lucarelli E Sangiorgi L Maini V Lattanzi G Marmiroli S Reggiani M. Troglitazione affects survival of human osteosarcoma cells. Int J Cancer. (2002) 98:344–51. doi: 10.1002/ijc.10203 11920584

[211] Vella S Conaldi PG Florio T Pagano A. PPAR gamma in neuroblastoma: the translational perspectives of hypoglycemic drugs. PPAR Res. (2016) 2016:3038164. doi: 10.1155/2016/3038164 27799938 PMC5069360

[212] Peri A Cellai I Benvenuti S Luciani P Baglioni S Serio M. PPARgamma in neuroblastoma. PPAR Res. (2008) 2008:917815. doi: 10.1155/ppar.v2008.1 18528516 PMC2397449

[213] Lee JJ Drakaki A Iliopoulos D Struhl K. MiR-27b targets PPARγ to inhibit growth, tumor progression and the inflammatory response in neuroblastoma cells. Oncogene. (2012) 31:3818–25. doi: 10.1038/onc.2011.543 PMC3290753 22120719

[214] Jiang W Cai X Xu T Liu K Yang D Fan L. Tripartite motif-containing 46 promotes viability and inhibits apoptosis of osteosarcoma cells by activating NF-B signaling through ubiquitination of PPAR. Oncol Res. (2020) 28:409–21. doi: 10.3727/096504020X15868639303417 PMC7851538 32295675

[215] Hauser S Adelmant G Sarraf P Wright HM Mueller E Spiegelman BM. Degradation of the peroxisome proliferator-activated receptor gamma is linked to ligand-dependent activation. J Biol Chem. (2000) 275:18527–33. doi: 10.1074/jbc.M001297200 10748014

[216] Kilroy G Kirk-Ballard LE Carter LE Floyd ZE. The ubiquitin ligase Siah2 regulates PPARγ activity in adipocytes. Endocrinology. (2012) 153:1206–18. doi: 10.1210/en.2011-1725 PMC3281538 22294748

[217] Han L Wang P Zhao G Wang H Wang M Chen J. Upregulation of SIRT1 by 17β-estradiol depends on ubiquitin-proteasome degradation of PPAR-γ mediated by NEDD4-1. Protein Cell. (2013) 4:310–21. doi: 10.1007/s13238-013-2124-z PMC4703587 23549616

[218] Ning Z Guo X Liu X Lu C Wang A Wang X. USP22 regulates lipidome accumulation by stabilizing PPARγ in hepatocellular carcinoma. Nat Commun. (2022) 13:2187. doi: 10.1038/s41467-022-29846-9 35449157 PMC9023467

[219] Hou Y Moreau F Chadee K. PPARγ is an E3 ligase that induces the degradation of NFκB/p65. Nat Commun. (2012) 3:1300. doi: 10.1038/ncomms2270 23250430

[220] Sato Y Sasaki H Kobayashi Y Haruki N Toyama T Kondo S. Expression of PPAR-gamma is correlated with the clinical course of neuroblastoma. J Pediatr Surg. (2003) 38:205–10. doi: 10.1053/jpsu.2003.50044 12596104

[221] Lee JH Lee GY Jang H Choe SS Koo SH Kim JB. Ring finger protein20 regulates hepatic lipid metabolism through protein kinase A-dependent sterol regulatory element binding protein1c degradation. Hepatology. (2014) 60:844–57. doi: 10.1002/hep.27011 PMC4258077 24425205

[222] Esquejo RM Jeon TI Osborne TF. Lipid-cell cycle nexus: SREBP regulates microRNAs targeting Fbxw7. Cell Cycle. (2014) 13:339–40. doi: 10.4161/cc.27509 PMC3956523 24335356

[223] Jeon TI Esquejo RM Roqueta-Rivera M Phelan PE Moon YA Govindarajan SS. An SREBP-responsive microRNA operon contributes to a regulatory loop for intracellular lipid homeostasis. Cell Metab. (2013) 18:51–61. doi: 10.1016/j.cmet.2013.06.010 23823476 PMC3740797

[224] Giandomenico V Simonsson M Grönroos E Ericsson J. Coactivator-dependent acetylation stabilizes members of the SREBP family of transcription factors. Mol Cell Biol. (2003) 23:2587–99. doi: 10.1128/MCB.23.7.2587-2599.2003 PMC150731 12640139

[225] Lee JP Brauweiler A Rudolph M Hooper JE Drabkin HA Gemmill RM. The TRC8 ubiquitin ligase is sterol regulated and interacts with lipid and protein biosynthetic pathways. Mol Cancer Res. (2010) 8:93–106. doi: 10.1158/1541-7786.MCR-08-0491 20068067 PMC3086825

[226] Walker AK Yang F Jiang K Ji JY Watts JL Purushotham A. Conserved role of SIRT1 orthologs in fasting-dependent inhibition of the lipid/cholesterol regulator SREBP. Genes Dev. (2010) 24:1403–17. doi: 10.1101/gad.1901210 PMC2895199 20595232

[227] Bakan I Laplante M. Connecting mTORC1 signaling to SREBP-1 activation. Curr Opin Lipidol. (2012) 23:226–34. doi: 10.1097/MOL.0b013e328352dd03 22449814

[228] Xie W Wang J Tian S Zhao H Cao L Liang Z. RNF126-mediated ubiquitination of FSP1 affects its subcellular localization and ferroptosis. Oncogene. (2024) 43:1463–75. doi: 10.1038/s41388-024-02949-x 38514855

[229] Liu W Zheng M Zhang R Jiang Q Du G Wu Y. RNF126-mediated MRE11 ubiquitination activates the DNA damage response and confers resistance of triple-negative breast cancer to radiotherapy. Adv Sci (Weinh). (2023) 10:e2203884. doi: 10.1002/advs.202203884 36563124 PMC9929257

[230] Liu Y Jiang N Chen W Zhang W Shen X Jia B. TRIM59-mediated ferroptosis enhances neuroblastoma development and chemosensitivity through p53 ubiquitination and degradation. Heliyon. (2024) 10:e26014. doi: 10.1016/j.heliyon.2024.e26014 38434050 PMC10906161

[231] Yoshida GJ. Emerging roles of Myc in stem cell biology and novel tumor therapies. J Exp Clin Cancer Res. (2018) 37:173. doi: 10.1186/s13046-018-0835-y 30053872 PMC6062976

[232] Ohya S Kajikuri J Endo K Kito H Elboray EE Suzuki T. Ca(2+) -activated K(+) channel K(Ca) 1.1 as a therapeutic target to overcome chemoresistance in three-dimensional sarcoma spheroid models. Cancer Sci. (2021) 112:3769–83. doi: 10.1111/cas.v112.9 PMC8409426 34181803

[233] Fahy E Subramaniam S Murphy RC Nishijima M Raetz CR Shimizu T. Update of the LIPID MAPS comprehensive classification system for lipids. J Lipid Res. (2009) 50 Suppl:S9–14. doi: 10.1194/jlr.R800095-JLR200 19098281 PMC2674711

[234] Suvarna K Jayabal P Ma X Wang H Chen Y Weintraub ST. Ceramide-induced cleavage of GPR64 intracellular domain drives Ewing sarcoma. Cell Rep. (2024) 43:114497. doi: 10.1016/j.celrep.2024.114497 39024100 PMC11416865

[235] Nikolaenko V Warnock D Mills K Heywood WE. Elucidating the toxic effect and disease mechanisms associated with Lyso-Gb3 in Fabry disease. Hum Mol Genet. (2023) 32:2464–72. doi: 10.1093/hmg/ddad073 PMC10360391 37145097

[236] Yin P Tang M Zhao G. M2 macrophage exosome-derived Apoc1 promotes ferroptosis resistance in osteosarcoma by inhibiting ACSF2 deubiquitination. Mol Carcinog. (2024) 63:2103–18. doi: 10.1002/mc.v63.11 39041949

[237] Wu F Zhang C Zhao C Wu H Teng Z Jiang T. Prostaglandin E1 inhibits GLI2 amplification-associated activation of the hedgehog pathway and drug refractory tumor growth. Cancer Res. (2020) 80:2818–32. doi: 10.1158/0008-5472.CAN-19-2052 32371475

[238] Knowles LM Axelrod F Browne CD Smith JW. A fatty acid synthase blockade induces tumor cell-cycle arrest by down-regulating Skp2. J Biol Chem. (2004) 279:30540–5. doi: 10.1074/jbc.M405061200 15138278

[239] Merklinger L Bauer J Pedersen PA Damgaard RB Morth JP. Phospholipids alter activity and stability of mitochondrial membrane-bound ubiquitin ligase MARCH5. Life Sci Alliance. (2022) 5. doi: 10.26508/lsa.202101309 PMC9034062 35459736

[240] Liu R Zeng LW Li HF Shi JG Zhong B Shu HB. PD-1 signaling negatively regulates the common cytokine receptor γ chain via MARCH5-mediated ubiquitination and degradation to suppress anti-tumor immunity. Cell Res. (2023) 33:923–39. doi: 10.1038/s41422-023-00890-4 PMC10709454 37932447

[241] Sakamaki JI Ode KL Kurikawa Y Ueda HR Yamamoto H Mizushima N. Ubiquitination of phosphatidylethanolamine in organellar membranes. Mol Cell. (2022) 82:3677–3692.e11. doi: 10.1016/j.molcel.2022.08.008 36044902

[242] He Y Wang J Xiao T. Targeting the ubiquitin-proteasome system: a novel therapeutic strategy for neuroblastoma. Front Oncol. (2024) 14:1443256. doi: 10.3389/fonc.2024.1443256 39391247 PMC11464458

[243] Senft D Qi J Ronai ZA. Ubiquitin ligases in oncogenic transformation and cancer therapy. Nat Rev Cancer. (2018) 18:69–88. doi: 10.1038/nrc.2017.105 29242641 PMC6054770

[244] Deng L Meng T Chen L Wei W Wang P. The role of ubiquitination in tumorigenesis and targeted drug discovery. Signal Transduct Target Ther. (2020) 5:11. doi: 10.1038/s41392-020-0107-0 32296023 PMC7048745

[245] Mansour MA. Ubiquitination: Friend and foe in cancer. Int J Biochem Cell Biol. (2018) 101:80–93. doi: 10.1016/j.biocel.2018.06.001 29864543

[246] Konopleva M Martinelli G Daver N Papayannidis C Wei A Higgins B. MDM2 inhibition: an important step forward in cancer therapy. Leukemia. (2020) 34:2858–74. doi: 10.1038/s41375-020-0949-z 32651541

[247] Montagut AM Armengol M de Pablo GG Estrada-Tejedor R Borrell JI Roué G. Recent advances in the pharmacological targeting of ubiquitin-regulating enzymes in cancer. Semin Cell Dev Biol. (2022) 132:213–29. doi: 10.1016/j.semcdb.2022.02.007 35184940

[248] Fricker LD. Proteasome inhibitor drugs. Annu Rev Pharmacol Toxicol. (2020) 60:457–76. doi: 10.1146/annurev-pharmtox-010919-023603 31479618

[249] Loi M Becherini P Emionite L Giacomini A Cossu I Destefanis E. sTRAIL coupled to liposomes improves its pharmacokinetic profile and overcomes neuroblastoma tumour resistance in combination with Bortezomib. J Control Release. (2014) 192:157–66. doi: 10.1016/j.jconrel.2014.07.009 25041999

[250] Yang F Jove V Chang S Hedvat M Liu L Buettner R. Bortezomib induces apoptosis and growth suppression in human medulloblastoma cells, associated with inhibition of AKT and NF-ĸB signaling, and synergizes with an ERK inhibitor. Cancer Biol Ther. (2012) 13:349–57. doi: 10.1016/j.jconrel.2014.07.009 PMC3341212 22313636

[251] Brignole C Marimpietri D Pastorino F Nico B Di Paolo D Cioni M. Effect of bortezomib on human neuroblastoma cell growth, apoptosis, and angiogenesis. J Natl Cancer Inst. (2006) 98:1142–57. doi: 10.1093/jnci/djj309 16912267

[252] Adams J Kauffman M. Development of the proteasome inhibitor Velcade (Bortezomib). Cancer Invest. (2004) 22:304–11. doi: 10.1093/jnci/djj309 15199612

[253] Adams J Palombella VJ Sausville EA Johnson J Destree A Lazarus DD. Proteasome inhibitors: a novel class of potent and effective antitumor agents. Cancer Res. (1999) 59:2615–22. doi: 10.1081/cnv-120030218 10363983

[254] Groll M Berkers CR Ploegh HL Ovaa H. Crystal structure of the boronic acid-based proteasome inhibitor bortezomib in complex with the yeast 20S proteasome. Structure. (2006) 14:451–6. doi: 10.1016/j.str.2005.11.019 16531229

[255] Kuhn DJ Chen Q Voorhees PM Strader JS Shenk KD Sun CM. Potent activity of carfilzomib, a novel, irreversible inhibitor of the ubiquitin-proteasome pathway, against preclinical models of multiple myeloma. Blood. (2007) 110:3281–90. doi: 10.1182/blood-2007-01-065888 PMC2200918 17591945

[256] Herndon TM Deisseroth A Kaminskas E Kane RC Koti KM Rothmann MD. U.s. Food and Drug Administration approval: carfilzomib for the treatment of multiple myeloma. Clin Cancer Res. (2013) 19:4559–63. doi: 10.1158/1078-0432.CCR-13-0755 23775332

[257] Lee EC Fitzgerald M Bannerman B Donelan J Bano K Terkelsen J. Antitumor activity of the investigational proteasome inhibitor MLN9708 in mouse models of B-cell and plasma cell Malignancies. Clin Cancer Res. (2011) 17:7313–23. doi: 10.1158/1078-0432.CCR-11-0636 PMC3443972 21903769

[258] Augello G Modica M Azzolina A Puleio R Cassata G Emma MR. Preclinical evaluation of antitumor activity of the proteasome inhibitor MLN2238 (ixazomib) in hepatocellular carcinoma cells. Cell Death Dis. (2018) 9:28. doi: 10.1038/s41419-017-0195-0 29348495 PMC5833482

[259] Morrow JK Lin HK Sun SC Zhang S. Targeting ubiquitination for cancer therapies. Future Med Chem. (2015) 7:2333–50. doi: 10.4155/fmc.15.148 PMC4976843 26630263

[260] Li Q Zhang W. Progress in anticancer drug development targeting ubiquitination-related factors. Int J Mol Sci. (2022) 23. doi: 10.3390/ijms232315104 PMC9737479 36499442

[261] Drexler HC. Activation of the cell death program by inhibition of proteasome function. Proc Natl Acad Sci U S A. (1997) 94:855–60. doi: 10.1073/pnas.94.3.855 PMC19603 9023346

[262] Grimm LM Goldberg AL Poirier GG Schwartz LM Osborne BA. Proteasomes play an essential role in thymocyte apoptosis. EMBO J. (1996) 15:3835–44. doi: 10.1002/j.1460-2075.1996.tb00757.x PMC452071 8670888

[263] Bobba A Canu N Atlante A Petragallo V Calissano P Marra E. Proteasome inhibitors prevent cytochrome c release during apoptosis but not in excitotoxic death of cerebellar granule neurons. FEBS Lett. (2002) 515:8–12. doi: 10.1016/S0014-5793(02)02231-7 11943185

[264] Butts BD Hudson HR Linseman DA Le SS Ryan KR Bouchard RJ. Proteasome inhibition elicits a biphasic effect on neuronal apoptosis via differential regulation of pro-survival and pro-apoptotic transcription factors. Mol Cell Neurosci. (2005) 30:279–89. doi: 10.1016/j.mcn.2005.07.011 16112871

[265] Lin KI Baraban JM Ratan RR. Inhibition versus induction of apoptosis by proteasome inhibitors depends on concentration. Cell Death Differ. (1998) 5:577–83. doi: 10.1038/sj.cdd.4400384 10200512

[266] Crawford LJ Walker B Irvine AE. Proteasome inhibitors in cancer therapy. J Cell Commun Signal. (2011) 5:101–10. doi: 10.1007/s12079-011-0121-7 PMC3088792 21484190

[267] Nardi IK Stark JM Larsen A Salgia R Raz DJ. USP22 interacts with PALB2 and promotes chemotherapy resistance via homologous recombination of DNA double-strand breaks. Mol Cancer Res. (2020) 18:424–35. doi: 10.1158/1541-7786.MCR-19-0053 PMC9285637 31685642

[268] Yadav P Subbarayalu P Medina D Nirzhor S Timilsina S Rajamanickam S. M6A RNA methylation regulates histone ubiquitination to support cancer growth and progression. Cancer Res. (2022) 82:1872–89. doi: 10.1158/0008-5472.CAN-21-2106 PMC9336196 35303054

[269] Zhang Q Zhu J Xie J Gu Y Chen L. USP22 as a key regulator of glycolysis pathway in osteosarcoma: insights from bioinformatics and experimental approaches. PeerJ. (2024) 12:e17397. doi: 10.7717/peerj.17397 38784391 PMC11114114

[270] Shan Q Yin L Zhan Q Yu J Pan S Zhuo J. The p-MYH9/USP22/HIF-1α axis promotes lenvatinib resistance and cancer stemness in hepatocellular carcinoma. Signal Transduct Target Ther. (2024) 9:249. doi: 10.1038/s41392-024-01963-5 39300073 PMC11412978

[271] Lu W Chu P Tang A Si L Fang D. The secoiridoid glycoside Gentiopicroside is a USP22 inhibitor with potent antitumor immunotherapeutic activity. BioMed Pharmacother. (2024) 177:116974. doi: 10.1016/j.biopha.2024.116974 38968798 PMC12772646

[272] Lu P Li Z Xu H. USP22 promotes gefitinib resistance and inhibits ferroptosis in non-small cell lung cancer by deubiquitination of MDM2. Thorac Cancer. (2024) 15:2260–71. doi: 10.1111/1759-7714.15439 PMC11543274 39315600

[273] Zhang Y Song J Zhou Y Jia H Zhou T Sun Y. Discovery of selective and potent USP22 inhibitors via structure-based virtual screening and bioassays exerting anti-tumor activity. Bioorg Chem. (2023) 141:106842. doi: 10.1016/j.bioorg.2023.106842 37769523

[274] Guo Y Zhang P Gao Z Liu X Su C Chen S. Inhibition of USP22 by miR-200b-5p represses gastric cancer cell proliferation and migration by targeting the NF-κB signaling pathway. Acta Biochim Biophys Sin (Shanghai). (2024). doi: 10.3724/abbs.2024231 PMC12247128 39711149

[275] Wang C Meng Y Zhao J Ma J Zhao Y Gao R. Deubiquitinase USP13 regulates glycolytic reprogramming and progression in osteosarcoma by stabilizing METTL3/m(6)A/ATG5 axis. Int J Biol Sci. (2023) 19:2289–303. doi: 10.7150/ijbs.82081 PMC10158027 37151889

[276] Kona SV Kalivendi SV. The USP10/13 inhibitor, spautin-1, attenuates the progression of glioblastoma by independently regulating RAF-ERK mediated glycolysis and SKP2. Biochim Biophys Acta Mol Basis Dis. (2024) 1870:167291. doi: 10.1016/j.bbadis.2024.167291 38857836

[277] Fang X Zhou W Wu Q Huang Z Shi Y Yang K. Deubiquitinase USP13 maintains glioblastoma stem cells by antagonizing FBXL14-mediated Myc ubiquitination. J Exp Med. (2017) 214:245–67. doi: 10.1084/jem.20151673 PMC5206492 27923907

[278] O'Brien DP Jones HBL Guenther F Murphy EJ England KS Vendrell I. Structural premise of selective deubiquitinase USP30 inhibition by small-molecule benzosulfonamides. Mol Cell Proteomics. (2023) 22:100609. doi: 10.1016/j.mcpro.2023.100609 37385347 PMC10400906

[279] Rusilowicz-Jones EV Jardine J Kallinos A Pinto-Fernandez A Guenther F Giurrandino M. USP30 sets a trigger threshold for PINK1-PARKIN amplification of mitochondrial ubiquitylation. Life Sci Alliance. (2020) 3. doi: 10.26508/lsa.202000768 PMC7362391 32636217

[280] Wang N Li J Xin Q Xu N. USP30-AS1 contributes to mitochondrial quality control in glioblastoma cells. Biochem Biophys Res Commun. (2021) 581:31–7. doi: 10.1016/j.bbrc.2021.10.006 34653676

[281] Wang B Wang X Du X Gao S Liang B Yao W. Identification and prognostic evaluation of differentially expressed long noncoding RNAs associated with immune infiltration in osteosarcoma. Heliyon. (2024) 10:e27023. doi: 10.1016/j.heliyon.2024.e27023 38463807 PMC10920385

[282] Nadolny C Zhang X Chen Q Hashmi SF Ali W Hemme C. Dysregulation and activities of ubiquitin specific peptidase 2b in the pathogenesis of hepatocellular carcinoma. Am J Cancer Res. (2021) 11:4746–67. doi: 10.1016/j.heliyon.2024.e27023.3 PMC8569343 34765291

[283] Liu D Fan Y Li J Cheng B Lin W Li X. Inhibition of cFLIP overcomes acquired resistance to sorafenib via reducing ER stress−related autophagy in hepatocellular carcinoma. Oncol Rep. (2018) 40:2206–14. doi: 10.3892/or.2018.6606 30066934

[284] Meng X Xiong Z Xiao W Yuan C Wang C Huang Y. Downregulation of ubiquitin-specific protease 2 possesses prognostic and diagnostic value and promotes the clear cell renal cell carcinoma progression. Ann Transl Med. (2020) 8:319. doi: 10.21037/atm.2020.02.141 32355763 PMC7186618

[285] Chen S Liu Y Zhou H. Advances in the development ubiquitin-specific peptidase (USP) inhibitors. Int J Mol Sci. (2021) 22. doi: 10.3390/ijms22094546 PMC8123678 33925279

[286] Davis MI Pragani R Fox JT Shen M Parmar K Gaudiano EF. Small molecule inhibition of the ubiquitin-specific protease USP2 accelerates cyclin D1 degradation and leads to cell cycle arrest in colorectal cancer and mantle cell lymphoma models. J Biol Chem. (2016) 291:24628–40. doi: 10.1074/jbc.M116.738567 PMC5114414 27681596

[287] Vora A Mitchell CD Lennard L Eden TO Kinsey SE Lilleyman J. Toxicity and efficacy of 6-thioguanine versus 6-mercaptopurine in childhood lymphoblastic leukaemia: a randomised trial. Lancet. (2006) 368:1339–48. doi: 10.1016/S0140-6736(06)69558-5 17046466

[288] Estlin EJ. Continuing therapy for childhood acute lymphoblastic leukaemia: clinical and cellular pharmacology of methotrexate, 6-mercaptopurine and 6-thioguanine. Cancer Treat Rev. (2001) 27:351–63. doi: 10.1053/ctrv.2002.0245 11908928

[289] Wang S Nie J Jiang H Li A Zhong N Tong W. VCP enhances autophagy-related osteosarcoma progression by recruiting USP2 to inhibit ubiquitination and degradation of FASN. Cell Death Dis. (2024) 15:788. doi: 10.1038/s41419-024-07168-6 39489738 PMC11532476

[290] Vriend J Glogowska A. Transcription of clock genes in medulloblastoma. Cancers (Basel). (2025) 17. doi: 10.3390/cancers17040575 PMC11852889 40002179

[291] Brockmann M Poon E Berry T Carstensen A Deubzer HE Rycak L. Small molecule inhibitors of aurora-a induce proteasomal degradation of N-myc in childhood neuroblastoma. Cancer Cell. (2013) 24:75–89. doi: 10.1016/j.ccr.2013.05.005 23792191 PMC4298657

[292] Wang Z Song Q Xue J Zhao Y Qin S. Ubiquitin-specific protease 28 is overexpressed in human glioblastomas and contributes to glioma tumorigenicity by regulating MYC expression. Exp Biol Med (Maywood). (2016) 241:255–64. doi: 10.1177/1535370215595468 PMC4935444 26209720

[293] Melino G Gallagher E Aqeilan RI Knight R Peschiaroli A Rossi M. Itch: a HECT-type E3 ligase regulating immunity, skin and cancer. Cell Death Differ. (2008) 15:1103–12. doi: 10.1038/cdd.2008.60 18552861

[294] Bernassola F Karin M Ciechanover A Melino G. The HECT family of E3 ubiquitin ligases: multiple players in cancer development. Cancer Cell. (2008) 14:10–21. doi: 10.1016/j.ccr.2008.06.001 18598940

[295] Hansen TM Rossi M Roperch JP Ansell K Simpson K Taylor D. Itch inhibition regulates chemosensitivity *in vitro* . Biochem Biophys Res Commun. (2007) 361:33–6. doi: 10.1016/j.bbrc.2007.06.104 17640619

[296] Nicolai S Pieraccioli M Peschiaroli A Melino G Raschellà G. Neuroblastoma: oncogenic mechanisms and therapeutic exploitation of necroptosis. Cell Death Dis. (2015) 6:e2010. doi: 10.1038/cddis.2015.354 26633716 PMC4720889

[297] Bongiorno-Borbone L Giacobbe A Compagnone M Eramo A De Maria R Peschiaroli A. Anti-tumoral effect of desmethylclomipramine in lung cancer stem cells. Oncotarget. (2015) 6:16926–38. doi: 10.18632/oncotarget.v6i19 PMC4627282 26219257

[298] Rossi M Rotblat B Ansell K Amelio I Caraglia M Misso G. High throughput screening for inhibitors of the HECT ubiquitin E3 ligase ITCH identifies antidepressant drugs as regulators of autophagy. Cell Death Dis. (2014) 5(5):e1203. doi: 10.1038/cddis.2014.113 24787015 PMC4047876

[299] Huan X Li J Chu Z Zhang H Cheng L Lun P. Dysregulation of iron homeostasis mediated by FTH increases ferroptosis sensitivity in TP53-mutant glioblastoma. Neurosci Bull. (2024). doi: 10.1007/s12264-024-01322-y PMC11978602 39666195

[300] Le Clorennec C Subramonian D Huo Y Zage PE. UBE4B interacts with the ITCH E3 ubiquitin ligase to induce Ku70 and c-FLIPL polyubiquitination and enhanced neuroblastoma apoptosis. Cell Death Dis. (2023) 14:739. doi: 10.1038/s41419-023-06252-7 37957138 PMC10643674

[301] Meng J Tagalakis AD Hart SL. Silencing E3 Ubiqutin ligase ITCH as a potential therapy to enhance chemotherapy efficacy in p53 mutant neuroblastoma cells. Sci Rep. (2020) 10:1046. doi: 10.1038/s41598-020-57854-6 31974512 PMC6978385

[302] Quirit JG Lavrenov SN Poindexter K Xu J Kyauk C Durkin KA. Indole-3-carbinol (I3C) analogues are potent small molecule inhibitors of NEDD4–1 ubiquitin ligase activity that disrupt proliferation of human melanoma cells. Biochem Pharmacol. (2017) 127:13–27. doi: 10.1016/j.bcp.2016.12.007 27979631

[303] Chuang HY Hsu LY Pan CM Pikatan NW Yadav VK Fong IH. The E3 ubiquitin ligase NEDD4–1 mediates temozolomide-resistant glioblastoma through PTEN attenuation and redox imbalance in nrf2-HO-1 axis. Int J Mol Sci. (2021) 22. doi: 10.3390/ijms221910247 PMC8549703 34638586

[304] Chen B Chen H Lu S Zhu X Que Y Zhang Y. KDM5B promotes tumorigenesis of Ewing sarcoma via FBXW7/CCNE1 axis. Cell Death Dis. (2022) 13:354. doi: 10.1038/s41419-022-04800-1 35428764 PMC9012801

[305] Grafals-Ruiz N Sánchez-Álvarez AO Santana-Rivera Y Lozada-Delgado EL Rabelo-Fernandez RJ Rios-Vicil CI. MicroRNA-92b targets tumor suppressor gene FBXW7 in glioblastoma. Front Oncol. (2023) 13:1249649. doi: 10.3389/fonc.2023.1249649 37752997 PMC10518455

[306] Zhang G Zhu Q Fu G Hou J Hu X Cao J. TRIP13 promotes the cell proliferation, migration and invasion of glioblastoma through the FBXW7/c-MYC axis. Br J Cancer. (2019) 121:1069–78. doi: 10.1038/s41416-019-0633-0 PMC6964669 31740732

[307] Zhang J Luo X Guo C Dai Z Tang X Zhang F. LncRNA GClnc1 promotes osteosarcoma progression by stabilizing NONO and blocking FBXW7-mediated ubiquitination. BMC Cancer. (2024) 24:1375. doi: 10.1186/s12885-024-13138-0 39523321 PMC11552323

[308] Lu B Feng Z Fan B Shi Y. Blocking miR-27a-3p sensitises Taxol resistant osteosarcoma cells through targeting Fbxw7. Bull Cancer. (2021) 108:596–604. doi: 10.1016/j.bulcan.2021.01.006 33863546

[309] Wang J Li T Wang B. Exosomal transfer of miR−25−3p promotes the proliferation and temozolomide resistance of glioblastoma cells by targeting FBXW7. Int J Oncol. (2021) 59. doi: 10.3892/ijo.2021.5244 PMC8295027 34278448

[310] Proto MC Fiore D Piscopo C Laezza C Bifulco M Gazzerro P. Modified adenosines sensitize glioblastoma cells to temozolomide by affecting DNA methyltransferases. Front Pharmacol. (2022) 13:815646. doi: 10.3389/fphar.2022.815646 35559231 PMC9086827

[311] Huang GD Cui P Ma GX Chen FF Chen ZB Li XJ. Phragmunis a suppresses glioblastoma through the regulation of MCL1-FBXW7 by blocking ELK1-SRF complex-dependent transcription. Neurochem Int. (2021) 147:105051. doi: 10.1016/j.neuint.2021.105051 33979572

[312] Orlicky S Tang X Neduva V Elowe N Brown ED Sicheri F. An allosteric inhibitor of substrate recognition by the SCF(Cdc4) ubiquitin ligase. Nat Biotechnol. (2010) 28:733–7. doi: 10.1038/nbt.1646 PMC4445864 20581844

[313] Principi E Sondo E Bianchi G Ravera S Morini M Tomati V. Targeting of ubiquitin E3 ligase RNF5 as a novel therapeutic strategy in neuroectodermal tumors. Cancers (Basel). (2022) 14. doi: 10.3390/cancers14071802 PMC8997491 35406574

[314] Rudzinski ER Anderson JR Lyden ER Bridge JA Barr FG Gastier-Foster JM. Myogenin, AP2β, NOS-1, and HMGA2 are surrogate markers of fusion status in rhabdomyosarcoma: a report from the soft tissue sarcoma committee of the children's oncology group. Am J Surg Pathol. (2014) 38:654–9. doi: 10.1097/PAS.0000000000000195 PMC4010390 24618610

[315] Shen J Wu Y Ruan W Zhu F Duan S. miR-1908 dysregulation in human cancers. Front Oncol. (2022) 12:857743. doi: 10.3389/fonc.2022.857743 35463352 PMC9021824

[316] Wang X Li X Tan L Zhang F Zhang J Zhao X. Identification and validation of lipid metabolism gene FASN-associated miRNA in wilms tumor. Biochem Genet. (2024). doi: 10.1007/s10528-024-10703-x 38416272

[317] Wang TF Wang H Peng AF Luo QF Liu ZL Zhou RP. Inhibition of fatty acid synthase suppresses U-2 OS cell invasion and migration via downregulating the activity of HER2/PI3K/AKT signaling pathway *in vitro* . Biochem Biophys Res Commun. (2013) 440:229–34. doi: 10.1016/j.bbrc.2013.09.024 24041695

[318] Liu ZL Wang G Peng AF Luo QF Zhou Y Huang SH. Fatty acid synthase expression in osteosarcoma and its correlation with pulmonary metastasis. Oncol Lett. (2012) 4:878–82. doi: 10.3892/ol.2012.862 PMC3499607 23162615

[319] Mao JH Zhou RP Peng AF Liu ZL Huang SH Long XH. microRNA-195 suppresses osteosarcoma cell invasion and migration *in vitro* by targeting FASN. Oncol Lett. (2012) 4:1125–9. doi: 10.3892/ol.2012.863 PMC3499598 23162665

[320] Long XH Mao JH Peng AF Zhou Y Huang SH Liu ZL. Tumor suppressive microRNA-424 inhibits osteosarcoma cell migration and invasion via targeting fatty acid synthase. Exp Ther Med. (2013) 5:1048–52. doi: 10.3892/etm.2013.959 PMC3628901 23599729

[321] Fan H Huang W Guo Y Ma X Yang J. α-linolenic acid suppresses proliferation and invasion in osteosarcoma cells via inhibiting fatty acid synthase. Molecules. (2022) 27. doi: 10.3390/molecules27092741 PMC9105512 35566090

[322] Huang KC Chuang PY Hsieh RZ Chen CN Chang SF Su YP. Stearoyl-coA desaturase-1 attenuates the high shear force damage effect on human MG63 osteosarcoma cells. Int J Mol Sci. (2020) 21. doi: 10.3390/ijms21134720 PMC7369751 32630668

[323] Ye M Gao R Chen S Bai J Chen J Lu F. FAM201A encodes small protein NBASP to inhibit neuroblastoma progression via inactivating MAPK pathway mediated by FABP5. Commun Biol. (2023) 6:714. doi: 10.1038/s42003-023-05092-7 37438449 PMC10338675

[324] Li J Zhu K Gu A Zhang Y Huang S Hu R. Feedback regulation of ubiquitination and phase separation of HECT E3 ligases. Proc Natl Acad Sci U S A. (2023) 120:e2302478120. doi: 10.1073/pnas.2302478120 37549262 PMC10438380

[325] Oyefiade A Erdman L Goldenberg A Malkin D Bouffet E Taylor MD. PPAR and GST polymorphisms may predict changes in intellectual functioning in medulloblastoma survivors. J Neurooncol. (2019) 142:39–48. doi: 10.1007/s11060-018-03083-x 30607709

[326] Urbanska K Pannizzo P Grabacka M Croul S Del Valle L Khalili K. Activation of PPARalpha inhibits IGF-I-mediated growth and survival responses in medulloblastoma cell lines. Int J Cancer. (2008) 123:1015–24. doi: 10.1002/ijc.v123:5 PMC3222922 18546270

[327] Bell E Ponthan F Whitworth C Westermann F Thomas H Redfern CP. Cell survival signalling through PPARδ and arachidonic acid metabolites in neuroblastoma. PloS One. (2013) 8:e68859. doi: 10.1371/journal.pone.0068859 23874790 PMC3706415

[328] Valentiner U Carlsson M Erttmann R Hildebrandt H Schumacher U. Ligands for the peroxisome proliferator-activated receptor-gamma have inhibitory effects on growth of human neuroblastoma cells *in vitro* . Toxicology. (2005) 213:157–68. doi: 10.1016/j.tox.2005.05.024 16009482

[329] Tang W Thundyil J Lim GGY Tng TJW Yeow SQZ Nair A. Parkin regulates neuronal lipid homeostasis through SREBP2-lipoprotein lipase pathway-implications for Parkinson's disease. Hum Mol Genet. (2023) 32:1466–82. doi: 10.1093/hmg/ddac297 PMC10117165 36519761

[330] Sun C Zhu G Shen C Huang S Li R Li J. Identification and validation of PCSK9 as a prognostic and immune-related influencing factor in tumorigenesis: a pan-cancer analysis. Front Oncol. (2023) 13:1134063. doi: 10.3389/fonc.2023.1134063 37860186 PMC10584329

